# NCOR2 represses MHC class I molecule expression to drive metastatic progression of breast cancer

**DOI:** 10.1038/s41467-026-72168-3

**Published:** 2026-05-05

**Authors:** Pavla Ticha, Jason J. Northey, Radhika Narain, Shivang Sharma, Johnathon N. Lakins, Hugo Gonzalez, Kelly Kersten, Alastair J. Ironside, Allison P. Drain, Martin Zidek, Kelvin K. Tsai, Yunn-Yi Chen, Eugene Shenderov, Valerie M. Weaver

**Affiliations:** 1 Department of Surgery and Center for Bioengineering and Tissue Regeneration, University of California, San Francisco, San Francisco, CA USA; 2 Department of Oncology, Johns Hopkins School of Medicine, Baltimore, MD USA; 3 Centro BASAL Ciencia & Vida FB210008, Fundación Ciencia & Vida, Santiago, Chile; 4 Facultad de Ciencias para el Cuidado de la Salud, Universidad San Sebastián, Santiago, Chile; 5 Department of Anatomy, University of California, San Francisco, San Francisco, CA USA; 6 Department of Pathology, University of California, San Francisco, San Francisco, CA USA; 7 Department of Pathology, Western General Hospital, NHS Lothian, Edinburgh, UK; 8 Laboratory of Advanced Molecular Therapeutics, Graduate Institute of Clinical Medicine, College of Medicine, Taipei Medical University, Taipei, Taiwan; 9 Department of Internal Medicine, Division of Gastroenterology, Wang Fang Hospital, Taipei Medical University, Taipei, Taiwan; 10 TMU Research Center of Cancer Translational Medicine, Taipei Medical University, Taipei, Taiwan; 11 The Bloomberg-Kimmel Institute for Cancer Immunotherapy, Johns Hopkins School of Medicine, Baltimore, MD USA; 12 Department of Radiation Oncology, University of California San Francisco, San Francisco, CA USA; 13 Department of Bioengineering and Therapeutic Sciences, University of California San Francisco, San Francisco, CA USA; 14 Eli and Edythe Broad Center of Regeneration Medicine and Stem Cell Research, University of California San Francisco, San Francisco, CA USA; 15 Helen Diller Family Comprehensive Cancer Center, University of California San Francisco, San Francisco, CA USA; 16 Present Address: Department of Plastic Surgery, 3rd Faculty of Medicine and University Hospital Kralovske Vinohrady, Charles University in Prague, Praha 10, Czech Republic; 17 Present Address: Department of Biochemistry, University of Alberta, Edmonton, AB Canada; 18 Present Address: Cancer Metabolism and Microenvironment Program, NCI-designated Cancer Center, Sanford Burnham Prebys Medical Discovery Institute, La Jolla, CA USA

**Keywords:** Breast cancer, Metastasis, Cancer microenvironment, Chromosomes, Tumour immunology

## Abstract

Metastatic progression depends upon the ability of disseminated tumor cells to evade immune surveillance. Major Histocompatibility Complex (MHC)-mediated antigen presentation facilitates T cell-dependent eradication of metastatic tumor cells. Here, we show that nuclear corepressor 2 (NCOR2) is an epigenetic regulator of MHC class I molecule presentation on breast tumor cells. Patients with triple negative breast cancer (TNBC) that express high levels of NCOR2 also exhibit reduced metastasis-free survival and decreased MHC class I expression, and the metastatic lesions in patients with TNBC have high nuclear NCOR2 and reduced Cluster of Differentiation (CD)8^+^ T cells. Reducing NCOR2 expression or preventing its interaction with Histone Deacetylase, HDAC3, enhances innate immune cell recruitment and activity, and elevates MHC class I levels on disseminated cancer cells to potentiate CD8^+^ T cell activity and apoptosis induction that prevents metastatic progression. The studies provide evidence to support NCOR2 as a targetable epigenetic regulator of metastasis towards which therapies could be developed to reduce patient mortality.

## Abstract (web summary)

Metastasis is often driven by immune suppression, including impaired MHC-I-mediated antigen presentation. This group identifies NCOR2 as an epigenetic repressor of MHC-I expression in aggressive breast cancer.

## Introduction

Cancer patient mortality is primarily due to treatment-resistant, metastatic disease^1^. Tumor cells can disseminate early and either remain dormant for years or generate progressive, metastatic disease^2–8^. Metastatic progression versus dormancy depends upon the growth and survival of the disseminated tumor cells (DTCs) at the metastatic site^9–14^, as well as the ability of the DTCs to evade immune surveillance^15–18^. Of these determinants, intrinsic tumor cell and extrinsic systemic and microenvironmental factors that regulate anti-tumor immunity have emerged as key regulators of tumor dormancy versus progression^2,3,9,16,19–29^. Clarifying mechanisms whereby disseminated tumor cells overcome antitumor immunity to generate progressive metastatic lesions is an area of intense investigation.

The expression of MHC class I molecules on the surface of cancer cells is a crucial step for antigen presentation and recognition by cytotoxic T cells^30–33^. While MHC molecules amplify the antitumor activity of T cells, MHC class I molecules primarily influence CD8 T cell activity, whereas MHC class II molecules influence CD4 T cell phenotypes^34–38^. Consistently, loss of MHC surface expression has been implicated in progressive metastatic disease^30^. Mechanisms regulating dysfunctional levels and function of MHC molecules in tumors include genetic mutations or polymorphisms in the MHC molecule or its accessory proteins^39^. These irreversible perturbations include downregulation or mutations/deletions in Human Leukocyte Antigen (HLA) alleles that reduce T cell recognition^40–42^, or accessory molecules that influence cell surface expression^43^, as well as polymorphisms that compromise tumor peptide binding and presentation^44,45^. By contrast, epigenetic silencing of MHC or associated processing molecules, modification of pathways that regulate transcription of MHC or its accessory molecules, including NFκB or interferon gamma (IFNγ) signaling^46^, or factors influencing post-translational processing, cell surface expression, and protein stability^47–50^, represent reversible mechanisms that could be targeted to potentiate antitumor immunity and eradicate metastatic burden.

Women with triple negative breast cancer (TNBC) and Human Epidermal growth factor Receptor 2-positive (HER2+ve) breast cancers exhibit high rates of progressive metastatic disease, despite successful primary tumor removal and comprehensive chemotherapy^51,52^. Developing targeted therapies to eradicate disseminated tumor cells with high potential to form metastatic lesions would go far towards enhancing breast cancer patient survival. We identified nuclear corepressor 2 (NCOR2) as an epigenetic regulator of treatment resistance in TNBC^53^. We showed that patients whose breast cancers express high levels of NCOR2 exhibit overall poor prognosis and reduced survival^53,54^. Mechanistically, we reported that NCOR2 represses apoptosis induction in response to chemotherapy, and that NCOR2 compromises immune checkpoint response by recruiting the Histone Deacetylase, HDAC3, to repress IRF-1 regulated expression of pro-apoptotic genes and tumor cell-secreted cytokines implicated in CD8+ T cell recruitment^53^. Given the link between treatment resistance, immune escape and metastasis, and evidence that IFNγ induces MHC expression, we hypothesized that NCOR2 may independently drive metastatic progression by epigenetically repressing MHC class I expression to enable immune evasion.

Here, we show a distinct and previously unrecognized function for NCOR2: the repression of MHC class 1 antigen presentation genes in disseminated tumor cells, thereby preventing immune detection and facilitating metastatic outgrowth in the lung. This work identifies NCOR2 as a key epigenetic regulator that mechanistically links immune evasion to metastatic progression and highlights it as a potential target to restore antitumor immunity.

## Results

### Nuclear NCOR2 is enriched in residual chemotherapy-treated human breast tumors

**Fig. 1:**
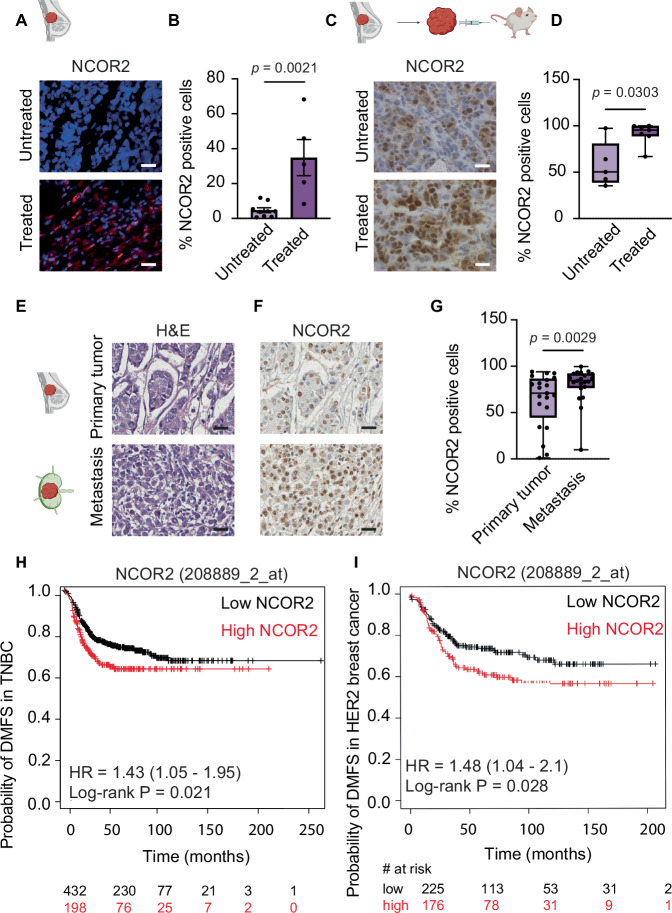
NCOR2 is enriched in residual chemotherapy treated human breast tumors and predicts reduced metastasis-free survival. **A** Representative images of frozen tissue sections from systemic chemotherapy treated and untreated human triple-negative breast cancer (TNBC) stained by immunofluorescence with an antibody specific to NCOR2. Nuclei are stained with DAPI. Scale bar: 20 μm. **B** Bar graphs showing quantification of positive NCOR2 staining from **A** (untreated patients, *n* = 9; treated patients, *n* = 5). **C** Representative images of formalin fixed paraffin embedded (FFPE) tissue sections from systemic chemotherapy treated and untreated TNBC patient-derived xenograft (PDX) primary tumors stained by immunohistochemistry (IHC) with an antibody specific to NCOR2. Nuclei are counterstained with hematoxylin. Scale bar: 20 μm. **D** Box and whisker plots showing quantification of positive NCOR2 IHC staining from **C** (untreated patients, *n* = 5; treated patients, *n* = 7). **E** Representative images of FFPE tissue sections from human primary ductal carcinomas and their matched lymph node metastases stained with hematoxylin and eosin (H&E). Scale bar: 20 μm. **F** Representative images of IHC staining with an antibody specific to NCOR2 using FFPE tissue sections as in **E**. Scale bar: 20 μm. **G** Box and whisker plots showing quantification of positive NCOR2 IHC staining from **F** (primary tumor, *n* = 21 patients; metastasis, *n* = 21 patients). **H** Kaplan–Meier plot (from http://kmplot.com) with hazard ratio (HR) and Log-rank displayed for the distant metastasis-free survival (DMFS) of TNBC patients partitioned by NCOR2 mRNA abundance in primary tumors (high vs. low; *n* = 630). **I** Kaplan–Meier plot (from http://kmplot.com) with hazard ratio (HR) and Log-rank displayed for the DMFS of HER2+ve breast cancer patients stratified as in **H** (high vs. low; *n* = 452). Bar graphs are represented as mean ± s.e.m using a two-sided unpaired *t*-test (**B**). Box and whisker plots are represented as median (centre line) and the interquartile range (box), whiskers indicate the minimum to maximum values. Statistical analysis was performed using a two-sided Mann–Whitney U test (**D**) or a two-sided Wilcoxon signed-rank test (**G**). Graphical elements were created with BioRender.com.

Approximately seventy percent of TNBCs that develop in patients exhibit a partial response to Neoadjuvant Chemotherapy (NAC) and retain some level of residual disease^55^. We previously implicated NCOR2 in systemic chemotherapy resistance in experimental and clinical TNBC^53^. Here, we began by asking whether NCOR2 expression in the residual TNBC primary tumors could explain their treatment resistance. Immunofluorescence (IF) analysis of the residual TNBCs excised from a cohort of patient biospecimens that had received NAC showed elevated nuclear NCOR2 levels compared to an independent cohort of non-treated TNBC tumors (Fig. 1A, B). Our findings are consistent with the possibility that NCOR2 contributed to the treatment resistance phenotype of these tumors.

To test whether chemotherapy treatment led to the enrichment of nuclear NCOR2 in therapy resistant human breast tumors, we next treated patient derived xenograft (PDX) TNBCs propagated orthotopically in NOD/SCID mice with Paclitaxel (10 mg/kg for 4 weeks, every second day starting 7 days after PDX injection), and thereafter stained the treated and untreated tissues by immunohistochemistry (IHC) to assess nuclear versus cytosolic NCOR2 protein. We quantified an increase in IHC staining for nuclear NCOR2 in the residual chemotherapy-treated resistant TNBC PDX tumors when compared to the cytosolic and heterogeneous nuclear levels of NCOR2 in the non-treated tumors (Fig. 1C, D). The data implicate NCOR2 in the residual treatment-resistant human TNBC phenotype.

**Fig. 2:**
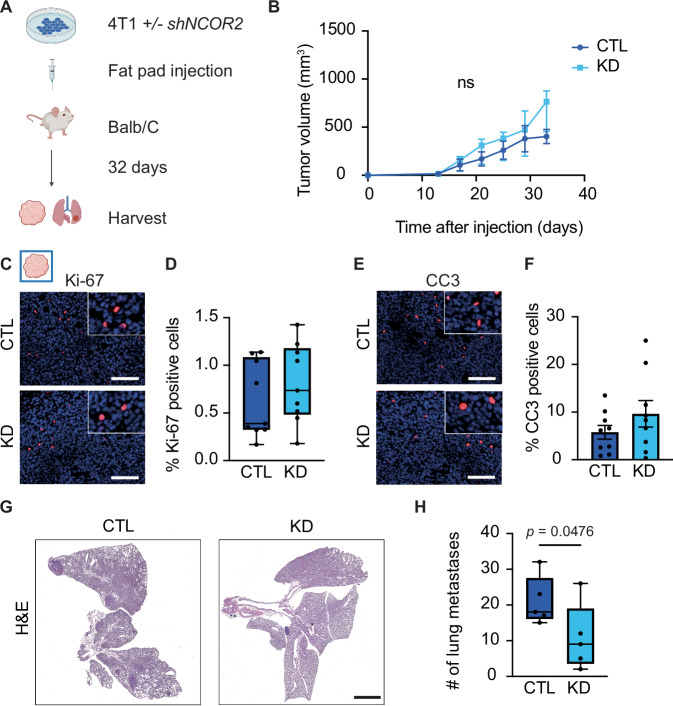
NCOR2 regulates lung metastasis from primary syngeneic mammary tumors. **A** Cartoon illustrating experimental design. **B** Mammary tumor outgrowth of 4T1 tumor cells injected into Balb/C mice with (KD) and without (CTL) NCOR2 knockdown (CTL, *n* = 9 mice; NCOR2 KD, *n* = 9 mice). **C** Representative images of immunofluorescence (IF) staining with an antibody specific to Ki-67 using frozen mammary tumor sections derived from the experimental mice in **B**. Scale bar: 100 μm. **D** Box and whisker plots showing quantification of the IF staining in **C** (CTL, *n* = 9 mice; NCOR2 KD, *n* = 9 mice). **E** Representative images of IF staining with an antibody specific to cleaved caspase-3 (CC3) using frozen mammary tumor sections derived from the experimental mice in **B**. Scale bar: 100 μm. **F** Bar graphs showing quantification of the IF staining in **E** (CTL, *n* = 9 mice; NCOR2 KD, *n* = 9 mice). **G** Representative images of hematoxylin and eosin (H&E) stained FFPE lung tissue sections taken from mice injected with 4T1 cells with (KD) and without (CTL) NCOR2 knockdown. Scale bar: 1 mm. **H** Box and whisker plots showing quantification of the number of lung metastases for each specimen processed as in **G** (CTL, *n* = 5 mice; NCOR2 KD, *n* = 5 mice). Line graph is represented as median and interquartile range using a time series analysis based on two-sided Mann–Whitney U tests (**B**). Box and whisker plots are represented as median (centre line) and interquartile range (box), whiskers indicate minimum to maximum values. Statistical analysis was performed using a two-sided Mann–Whitney U test (**D**) and one-sided Mann–Whitney U test (**H**). Bar graph is represented as mean ± s.e.m using a two-sided unpaired *t*-test (**F**). CTL control, KD knockdown, ns not significant. Graphical elements were created with BioRender.com.

### Metastatic human breast cancer cells are enriched in nuclear NCOR2, and high NCOR2 predicts lower metastasis-free survival in breast cancer patients

An incomplete pathological response to therapy is associated with worse overall survival in patients with TNBC^55^. TNBCs that fail to respond with a complete pathological response to chemotherapy frequently recur with metastatic disease that is refractory to multiple systemic therapies, with less than thirty percent of patients surviving beyond 5 years^56^. To determine whether there were links between NCOR2 and human breast cancer metastasis, we quantified the level of nuclear NCOR2 in tissue arrayed sections of human primary invasive TNBC ductal carcinoma and compared the findings to patient-matched lymph node metastasis. Quantitative IHC analysis revealed that there was a significant enrichment for nuclear NCOR2 in the tumor cells that had disseminated to the human lymph nodes in the patients with TNBC, as compared to the levels found in their patient matched primary invasive TNBC; out of the 21 pairs analyzed, 17 showed increased NCOR2 expression in the lymph node metastases compared to the primary tumor (Fig. 1E–G). In further support of an association between NCOR2 and reduced overall patient survival, distant metastasis-free survival (DMFS) of basal breast cancer patients stratified by NCOR2 mRNA abundance (high or low; see methods for criteria and statistical analysis) demonstrated that patients with high NCOR2 in their primary tumors had a less favorable DMFS (Fig. 1H). In addition, HER-2 positive breast cancer patients similarly stratified by NCOR2 mRNA abundance (high or low; see methods for criteria and statistical analysis) had a lower DMFS with high NCOR2 levels in their primary tumors (Fig. 1I). The findings implicate NCOR2 in breast cancer metastasis and extend the observations beyond TNBCs.

### NCOR2 regulates syngeneic mammary tumor lung metastasis

We next examined the role of NCOR2 in the metastasis of an orthotopic mouse model of breast cancer. We used the highly metastatic TNBC murine line 4T1, which expresses abundant NCOR2, and is derived from a tumor that developed within a Balb/C mouse that spontaneously forms progressive metastatic lesions in the liver, lung, brain, and bone of host syngeneic mice^57,58^. We employed two independent shRNA constructs and a scrambled control to knockdown NCOR2 levels in the 4T1 cells, and following confirmation of knockdown efficiency by immunoblot analysis (Supplementary Fig. 1A), these engineered cells were injected orthotopically into the mammary fat pads of syngeneic host mice (Fig. 2A). The studies revealed that NCOR2 expression had little to no impact on primary tumor growth (Fig. 2B). Moreover, IHC of the mammary tumor tissues excised at study termination revealed similar tumor cell proliferation and apoptosis levels, as indicated by staining for Ki-67 (Fig. 2C, D) and cleaved (activated) caspase 3 (Fig. 2E, F). Importantly, despite a lack of differences in primary tumor growth and survival, hematoxylin and eosin (H&E) analysis of corresponding sections from formalin fixed and parrafin embedded (FFPE) lung tissues revealed a significant reduction in the number of metastases in the mice injected with cells in which NCOR2 was knocked down, compared to those injected with 4T1 cells expressing a scrambled shRNA control (Fig. 2G, H). IHC analysis confirmed that the lung lesions that were derived from the 4T1 cells with NCOR2 knockdown maintained reduced NCOR2 levels as compared to 4T1 metastatic lesions expressing the scrambled shRNA control (Supplementary Fig. 1B, C). The findings suggest that reducing NCOR2 restricted the outgrowth of the metastatic lesions in the lungs. Consistently, H&E staining of the primary tumor tissue sections showed similar size, morphology, and histophenotype between the tumors with and without NCOR2 knockdown, implying NCOR2 did not repress metastasis by regulating the primary tumor phenotype, but rather tempered tumor metastasis either by influencing tumor cell dissemination or the metastatic outgrowth of the tumor cells after they reached the lungs.

### NCOR2 regulates lung metastasis from spontaneous mammary tumors

**Fig. 3:**
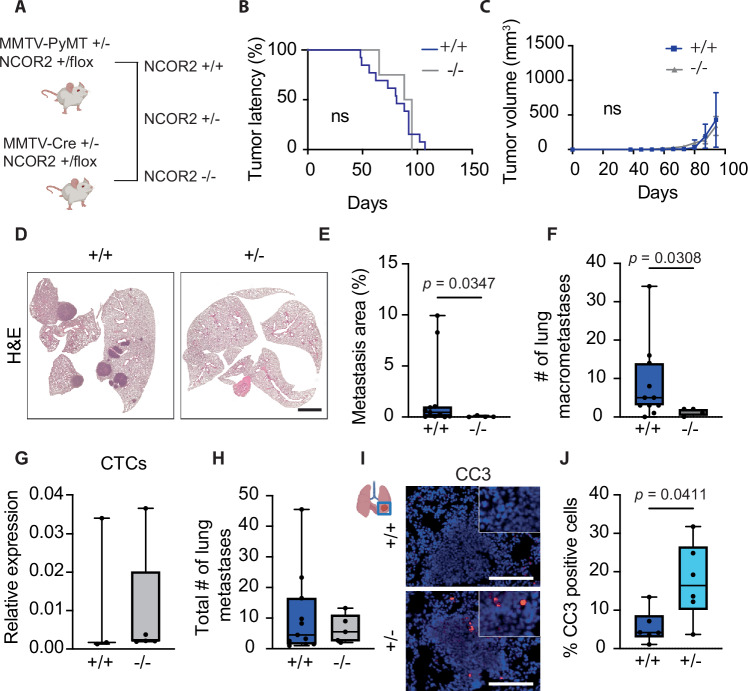
NCOR2 regulates lung metastasis in PyMT-NCOR2 knockout mice. **A** Cartoon illustrating the breeding scheme used to generate transgenic mice with polyomavirus middle T (MMTV-PyMT)-driven mammary tumors and conditional (MMTV-Cre induced) deletion of NCOR2 expression in mammary epithelial cells. **B** Survival plots displaying tumor latency (days) for MMTV-PyMT mice with wildtype levels (+/+) or knockout of NCOR2 (−/−) in mammary epithelial and tumor cells (PyMT-NCOR2^+/+^, *n* = 13 mice; PyMT-NCOR2^−/−^; *n* = 4 mice). **C** Mammary tumor outgrowth of the mouse cohorts described in (**B**) (PyMT-NCOR2^+/+^, *n* = 10 mice; PyMT-NCOR2^−/−^; *n* = 4 mice). **D** Representative images of hematoxylin and eosin (H&E)-stained FFPE tissue sections derived from the lungs of the mouse cohorts described in **B**. Scale bar: 1 mm. **E** Box and whisker plots showing quantification of the percentage of metastatic lesion area in relation to total lung area for the mice in **B** (PyMT-NCOR2^+/+^, *n* = 11 mice; PyMT-NCOR2^−/−^, *n* = 4 mice), **F** quantification of the number of lung macrometastases for the mice in **B** (PyMT-NCOR2^+/+^, *n* = 11 mice; PyMT-NCOR2^−/−^, *n* = 4 mice), **G** quantification of the relative levels of circulating tumor cells (CTCs) in the mouse cohorts as described in **B** (PyMT-NCOR2^+/+^, *n* = 3 mice; PyMT-NCOR2^−/−^, *n* = 5 mice), and **H** quantification of the total number of lung metastases for the mice in **B** (PyMT-NCOR2^+/+^, *n* = 11 mice; PyMT-NCOR2^−/−^, *n* = 5 mice). **I** Representative images showing IF staining with an antibody specific to cleaved caspase 3 (CC3) in FFPE lung tissue sections from PyMT-NCOR2^+/+^ and PyMT-NCOR2^+/-^ mice. Scale bar: 100 μm. **J** Box and whisker plots showing quantification of the IF staining in **I** (PyMT-NCOR2^+/+^, *n* = 6 mice; PyMT-NCOR2^+/^^−^, *n* = 6 mice). Line graps are represented as survival curves using a log-rank test **B** and a median and interquartile range using a time series analysis based on two-sided Mann–Whitney U tests (**C)**. Box and whisker plots are represented as median (centre line) and interquartile range (box), whiskers indicate minimum to maximum values. Statistical analysis was performed using a two-sided Mann–Whitney U test (**E**–**H**, **J**). ns: not significant.

To further explore the role of NCOR2 in breast cancer progression, we established cohorts of genetically engineered mice designed for the conditional deletion of NCOR2 from mammary epithelial cells (MMTV-Cre; -NCOR2^+/+^, -NCOR2^−/+^, and -NCOR2^−/−^) to assess the impact of NCOR2 levels on spontaneous polyomavirus middle T-driven (MMTV-PyMT) mammary tumor progression (Fig. 3A). After confirming genomic deletion and mammary-specific NCOR2 knockout by immunoblot and immunofluorescence in the mice (Supplementary Fig. 2A–D), cohorts of MMTV-PyMT-MMTV-Cre-NCOR2^+/+^; MMTV-PyMT-MMTV-Cre-NCOR2^+/^^−^; and MMTV-PyMT-MMTV-Cre-NCOR2^−/−^ mice were generated. The PyMT/NCOR2 mouse cohorts were then monitored for differences in spontaneous tumor formation and phenotype, circulating tumor cell (CTC) levels, and lung metastasis. Note: to simplify data presentation, we opted to summarize the findings for NCOR2^+/+^ and NCOR2^−/−^ cohorts for the majority of the assessed criteria within the primary figure, and the three cohort results together with relevant additional analysis within the Supplementary Figure. Primary tumor latency was not significantly different in all three mouse cohorts (Fig. 3B and Supplementary Fig. 2E), nor was there any change in tumor volume over time as determined by weekly caliper measurement (Fig. 3C and Supplementary Fig. 2F). Mouse survival, as indicated by the time when primary tumor size reached a pre-established end point of 1.5 cm in one diameter, and the number of tumors per mouse, also did not differ between experimental groups (Supplementary Fig. 2G, H). Histopathological examination by a certified pathologist of H&E-stained primary mammary tumor tissue sections indicated that wild-type control (+/+) and NCOR2 knockout (−/−) tumors were similar in histophenotype (Supplementary Fig. 2I). In each cohort, the tumors were moderately or poorly differentiated adenocarcinomas with papillary, trabecular, and acinar growth patterns showing varying degrees of differentiation (nuclei showed increased amount of mitotic activity and pleomorphism), with about a third of the tumors showing significant necrosis (>10%). Immunofluorescence analysis of primary excised tumors confirmed there were no significant differences in proliferation or apoptosis between mouse mammary tumor groups, as indicated by Ki-67 and activated caspase 3 positive levels, respectively (Supplementary Fig. 2J–M). The findings indicated that NCOR2 expression had little to no impact on the primary tumor histophenotype, growth, and apoptosis of endogenous mammary tumors. However, macroscopic examination of the lungs excised at study termination showed a reduced number of metastatic surface nodules in the PyMT NCOR2^−/−^ and PyMT NCOR2^−/+^ mice compared to the PyMT NCOR2^+/+^ mice (Supplementary Fig. 2N). Although histopathological examination by H&E staining of excised lung tissue sections suggested there was no significant reduction in total number of metastatic foci in the PyMT NCOR2^−/−^ or PyMT NCOR2^−/+^ mice relative to the wild type PyMT-NCOR2^+/+^ mice (Fig. 3D and Supplementary Fig. 2O), there was a significant decrease in metastatic foci size (Fig. 3E, Supplementary Fig. 2P). To distinguish between micro- and macrometastases, we used a size cut-off of 100 µm in the largest diameter (Supplementary Fig. 2Q). This threshold corresponds to the definition of early-stage metastatic lesions in preclinical models and is consistent with previously published research^59^. Further examination of lung tissues using this criteria demonstrated a higher number of macrometastases in the PyMT NCOR2^+/+^ mice (Fig. 3F and Supplementary Fig. 2R).

Importantly, CTC levels were not significantly different between the PyMT-NCOR2^+/+^ and PyMT NCOR2^−/−^ and PyMT NCOR2^−/+^ mouse cohorts (Fig. 3G and Supplementary Fig. 2S), suggesting NCOR2 had little impact on vascular dissemination of tumor cells from the primary site. Instead, the findings indicate that NCOR2 influenced the metastatic outgrowth of disseminated breast tumor cells after they reached their distal tissue. This finding is consistent with our inability to detect a difference in the total number of metastatic lesions (all micro- and macrometastases) between the experimental groups (Fig. 3H, and Supplementary Fig. 2T). Interestingly, although we quantified no differences in the proliferation (Ki67-positive) of the metastatic lesions (Supplementary Fig. 2U, V), we did observe a significant increase in apoptosis within the tumor cells that had disseminated to the lungs of the NCOR2 heterozygous mice compared to the NCOR2 control mice, as determined by immunostaining for activated caspase 3 (Fig. 3I, J). Note: we were only able to analyze proliferation and apoptosis of lung metastases in the NCOR2^+/^^−^ mouse cohort due to the scarcity of detectable lung metastatic lesions in the NCOR2^−/−^ mice. In conclusion, although NCOR2 appears to have little impact on primary tumor development and growth, our findings indicate that it strongly enhances the metastatic survival of disseminated tumor cells to foster their ability to form progressive metastatic lesions.

### NCOR2-HDAC3 interactions promote metastatic outgrowth of breast cancer cells

**Fig. 4:**
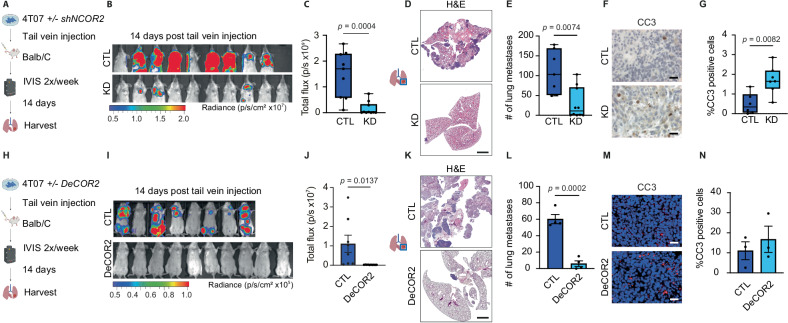
NCOR2 regulates lung metastatic outgrowth following tail vein injection. **A** Cartoon illustrating experimental design. **B** Representative bioluminescence images of Balb/C mice 14 days after tail vein injection with 4T07 cells with (KD, *n* = 10 mice) or without (CTL, *n* = 9 mice) knockdown of NCOR2. **C** Box and whisker plots showing quantification of the bioluminescence imaging (BLI) total radiance for the mice in **B** (CTL, *n* = 9 mice; KD, *n* = 10 mice). **D** Representative images of hematoxylin and eosin (H&E)-stained FFPE tissue sections derived from the lungs of the mice in **B**. Scale bar: 1 mm. **E** Box and whisker plots showing H&E determined quantification of the number of lung metastases for the mice in **B** (CTL, *n* = 7 mice; KD, *n* = 10 mice). **F** Representative images showing immunohistochemical (IHC) staining with an antibody specific to cleaved caspase 3 (CC3) in FFPE lung tissue sections from mice in **B**. Scale bar: 20 μm. **G** Box and whisker plots showing quantification of the IHC CC3 staining in **F** (CTL, *n* = 6 mice; KD, *n* = 7 mice). **H** Cartoon illustrating experimental design. **I** Representative bioluminescence images of Balb/C mice 14 days after tail vein injection with 4T07 cells expressing an empty vector (CTL, *n* = 8 mice) or the DeCOR2 peptide (*n* = 10 mice) that disrupts the NCOR2-HDAC3 interaction. **J** Bar graphs showing quantification of the BLI total radiance for the mice in **I** (CTL, *n* = 8 mice; DeCOR2, *n* = 10 mice). **K** Representative images of H&E-stained FFPE tissue sections derived from the lungs of the mice in **I**. Scale bar: 1 mm. **L** Box and whisker plots showing H&E determined quantification of the number of lung metastases for the mice in **I** (CTL, *n* = 4 mice; DeCOR2, *n* = 4 mice). **M** Representative images showing immunofluorescence (IF) staining with an antibody specific to CC3 in lung tissue sections from mice in **I**. Scale bar: 100 μm. **N** Bar graphs showing quantification of the IF CC3 staining in **M** (CTL, *n* = 3 mice; DeCOR2, *n* = 3 mice). Box and whisker plots are represented as median (centre line) and interquartile range (box), whiskers indicate minimum to maximum values. Statistical analysis was performed using a two-sided Mann–Whitney U test (**C**, **E**, **G**). Bar graphs are represented as mean ± s.e.m using a two-sided unpaired *t*-test (**J**, **L**, **N**). CTL control, KD knockdown. Graphical elements were created with BioRender.com.

To further explore the possibility that NCOR2 directly regulates the survival of disseminated tumor cells to favor their metastatic outgrowth, we exploited the less aggressive murine 4T07 breast cancer cell line. 4T07 breast cancer cells are unable to spontaneously form lung metastasis when injected into the mammary fat pad of host syngeneic mice. However, these tumor cells will form metastatic lung lesions when injected via tail vein^57^. After confirming that the 4T07 breast cancer cells expressed abundant NCOR2 (Supplementary Fig. 3A), we transduced the 4T07 breast cancer cells with the same lentiviral shRNA constructs we used to knockdown NCOR2 levels in the 4T1 cells, and verified similarly effective reduction of NCOR2 protein (Supplementary Fig. 3A). We then injected the tumor cells into the tail vein of host syngeneic mice and monitored the mice twice per week for metastatic lung growth using bioluminescence imaging (BLI) (Fig. 4A). Within seven days following tail vein injection, we were able to detect a BLI signal in the lungs of the mice that were injected with the 4T07 cells expressing the control shRNA (4T07-CTL), but little to no signal in the mice that had been injected with the cells in which NCOR2 had been knocked down (4T07-KD). Two weeks following tail vein injection, the BLI signal in 4T07-CTL injected mice was robust as compared to the much weaker signal for mice injected with the 4T07-KD cells (Fig. 4B, C). At study termination, H&E staining revealed a strikingly lower metastatic burden in the lungs of the mice that had been injected with 4T07-KD cells, as compared to mice receiving 4T07-CTL cells (Fig. 4D). Further analysis revealed a corresponding reduction in the number of detectable metastases and overall decreased metastatic lesion area for 4T07-KD cell derived lung metastases (Fig. 4E and Supplementary Fig. 3B). IHC analysis of FFPE lung tissue sections confirmed a reduction in NCOR2-positive staining in the lungs of mice injected with 4T07-KD cells (Supplementary Fig. 3C, D). IF staining of tissue sections to assess Ki-67-positivity of disseminated tumor cells revealed similar levels of proliferation in the lung metastases in both experimental groups (Supplementary Fig. 3E, F). However, a similar analysis of apoptosis in the few macrometastatic lung lesions derived from 4T07-KD cells with reduced NCOR2 levels that we could identify, uncovered higher levels of cell death in the metastatic lesions, as indicated by a more than threefold increase in cleaved caspase 3 positive IHC staining (Fig. 4F, G).

To clarify the molecular mechanisms regulating NCOR2-dependent metastatic outgrowth of 4T07 breast cancer cells, we perturbed NCOR2-HDAC3 interactions by expressing a decoy molecule termed DeCOR2 that we previously showed sequesters HDAC3, thereby preventing NCOR2-mediated epigenetic gene silencing in the 4T07 cells^53^. After verifying efficient expression of the myc-tagged DeCOR2 protein in infected 4T07 breast cancer cells by immunoblot and IF (Supplementary Fig. 3G, H), we injected these cells (4T07-DeCOR2) into the tail vein of host Balb/C mice and monitored their lung metastatic outgrowth as compared to vector control cells (4T07-CTL) as above (Fig. 4H). Similar to what was observed when NCOR2 was knocked down, preventing NCOR2-HDAC3 interactions and epigenetic silencing reduced lung metastasis as indicated by lower BLI signal 2 weeks post injection (Fig. 4I, J). Following lung excision, we observed a reduced number of visible metastatic foci on the surface of the lungs in the mice that were injected with 4T07-DeCOR2 cells, and by H&E staining of lung tissue sections, we quantified a significant corresponding reduction in the number and size of metastatic lesions (Fig. 4K, L and Supplementary Fig. 3I). Upon further analysis, once again, we detected more apoptosis in the lung lesions identified in the mice injected with 4T07-DeCOR2 cells, as indicated by elevated positive IF staining for cleaved caspase 3 (Fig. 4M, N). Notably, once again, the low sample size in this analysis was due to the overall low number of metastatic lesions detected in the lungs of mice injected with 4T07-DeCOR2 cells. The findings demonstrate that the HDAC3 repressor function of NCOR2 contributes to lung metastatic outgrowth by enhancing the survival of disseminated tumor cells.

### NCOR2 permits lung metastatic outgrowth by suppressing anti-tumor immunity

**Fig. 5:**
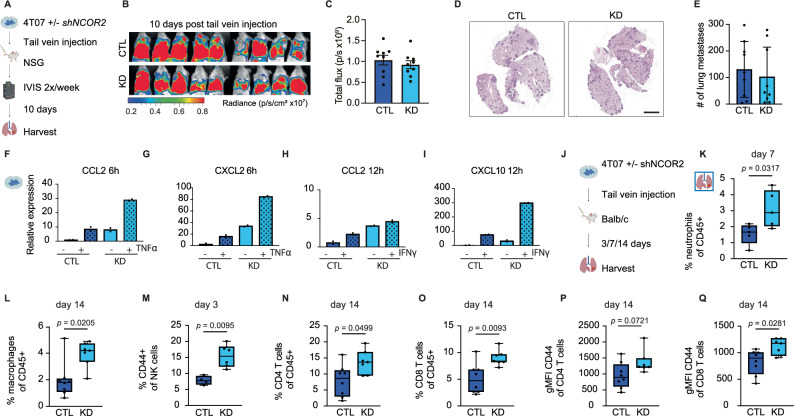
NCOR2 does not regulate lung metastasis in 4T07 tail vein injected immunodeficient mice. **A** Cartoon illustrating experimental design. **B** Representative bioluminescence images of NSG mice 10 days following tail vein injection with 4T07 cells with (KD, *n* = 9 mice) or without (CTL, *n* = 9 mice) knockdown of NCOR2. **C** Bar graphs showing quantification of BLI total radiance for the mice in **B** (CTL, *n* = 9 mice; KD, *n* = 9 mice). **D** Representative images of hematoxylin and eosin (H&E)-stained FFPE tissue sections derived from the lungs of the mice in **B**. Scale bar: 1 mm. **E** Bar graphs showing H&E determined quantification of the number of lung metastases for the mice in **B** (CTL, *n* = 9 mice; KD, *n* = 9 mice). **F** Bar graphs showing quantitative RT-PCR analysis of *Ccl2* expression relative to *Gapdh* for 4T07 cells with (KD, *n* = 2 independent experiments) or without (CTL, *n* = 2 independent experiments) knockdown of NCOR2 cultured and stimulated +/- TNFα for 6 h. **G** Analysis as in **F** for *Cxcl2* expression (CTL, *n* = 2 independent experiments; KD, *n* = 2 independent experiments). **H** Analysis as in (F) with the exception that cells were stimulated +/- IFNγ for 12 h (CTL, *n* = 2 independent experiments; KD, *n* = 2 independent experiments). **I** Analysis as in **H** for *Cxcl10* expression (CTL, *n* = 2 independent experiments; KD, *n* = 2 independent experiments). **J** Cartoon illustrating experimental design. **K** Box and whisker plots showing flow cytometry analysis to determine the percentage of CD45-positive cells that are neutrophils at 7 days following tail vein injection of 4T07 cells (CTL, *n* = 5 mice; KD *n* = 5 mice), **L** macrophages at 14 days following 4T07 cell tail vein injection (CTL, *n* = 8 mice; KD, *n* = 7 mice), **M** CD44-positive NK cells 3 days following tail vein injection of 4T07 cells (CTL, *n* = 4 mice; KD, *n* = 6 mice), and CD4+ (**N**) and CD8+ lymphocytes (**O**) at 14 days following 4T07 tail vein injection (CTL, *n* = 8 mice; KD, *n* = 7 mice). **P** Box and whisker plots showing flow cytometry analysis as in **N** to determine the gMFI of CD44 on the surface of CD4+ cells (CTL, *n* = 8 mice; KD, *n* = 7 mice) and **Q** as in **O** to determine the gMFI of CD44 on the surface of CD8+ T cells (CTL, *n* = 8 mice; KD, *n* = 7 mice). Bar graphs are represented as mean ± s.e.m. using a two-sided unpaired *t* test (**C**, **E**). Box and whisker plots are represented as median (centre line) and interquartile range (box), whiskers indicate minimum to maximum values. Statistical analysis was performed using a two-sided Mann–Whitney U test (**K**–**Q**). CTL control, KD knockdown. Graphical elements were created with BioRender.com.

Metastatic outgrowth is curtailed by anti-tumor immunity^60^. We and others previously demonstrated that NCOR2 represses the expression of multiple tumor secreted cytokines that stimulate CD8+ T cell recruitment^53,61,62^. To test whether NCOR2 permits metastatic outgrowth by repressing anti-tumor immunity, we repeated the 4T07 NCOR2 knockdown tail vein studies in a cohort of NOD SCID gamma (NSG) severely immunodeficient mice that lack both Natural Killer cells and T cells^63^ and monitored metastatic lung outgrowth by BLI (Fig. 5A). BLI revealed no significant differences in metastatic burden to the lungs between the two mouse cohorts (Fig. 5B, C), regardless of the level of NCOR2 expression in the tumor cells. Consistently, gross analysis of excised lung tissues revealed a similar metastatic burden between the two experimental groups, as indicated by cell surface foci (Fig. 5D), and quantification of metastatic lesions visualized by H&E staining of FFPE lung sections confirmed no significant differences in metastatic lesion number or size (Fig. 5E and Supplementary Fig. 4A). Moreover, IF and IHC staining of lung tissue sections conducted to examine levels of Ki67-positive and cleaved caspase 3-positive cells in the lungs of the host mice revealed similar levels of proliferation and apoptosis between the two groups (Supplementary Fig. 4B–E).

To study how NCOR2 knockdown affects anti-tumor immune responses, we evaluated cytokine expression in breast cancer cells. Prior studies showed that NCOR2 represses inflammation by inhibiting NFκB and STAT1 activity to reduce cytokine expression^53,61,64^. Consistently, we confirmed that NCOR2 represses interferon gamma (IFNγ)-induced induction of STAT1 in 4T07 cells (Supplementary Fig. 4F). We also noted that NCOR2 appeared to reduce the basal and tumor necrosis factor-alpha (TNFα)-induced expression of the proinflammatory cytokines CCL2, CXCL2 and CXCL5 (Fig. 5F, G, and Supplementary Fig. 4G), as well as IFNγ-dependent induction of CCL2 and CXCL10 (Fig. 5H, I) in the 4T07 tumor cells. To study the effect of these NCOR KD-mediated cytokine expression profiles on anti-tumor immunity, we used flow cytometry to analyze the immune cell composition in metastatic lungs on day 3, 7, and 14 after tail vein injection of NCOR2 KD and Control 4T07 cells (Fig. 5J). While CCL2 is well-known for its role in monocyte recruitment, we found no significant difference in CD11b+Ly6C+ monocytes between the lungs of mice bearing NCOR2 KD and control cells. However, in line with the upregulation of CXCL2 and CXCL5, we found a significant increase in the proportion of CD11b+Ly6G+ neutrophils on day 7 in NCOR2 KD inoculated lungs compared to controls (Fig. 5K and Supplementary Fig. 4H, I), findings that are consistent with prior studies, which have implicated a role for neutrophils in inhibiting metastasis^65–68^. We also noted a significant increase in the number of lung recruited macrophages on day 14 (Fig. 5L and Supplementary Fig. 4J). CXCL10 is known for its role in recruitment and activation of NK cells and T cells^69–73^. In line with this, we quantified a significant increase in CD44+NK cells in NCOR2 KD burdened lungs versus the control lungs (Fig. 5M and Supplementary Fig. 4K)^74–76^. Moreover, and consistent with the increased level of apoptosis we documented in the NCOR2 KD breast cancer cells, we documented a significant increase in the proportion of CD4 and CD8 T cells in the lungs of mice bearing NCOR2 KD cells (Fig. 5N, O and Supplementary Fig. 4L). These data suggest that NCOR2 in breast cancer cells represses innate cell and T cell recruitment to suppress anti-tumor immune responses in the metastatic lung. Importantly, we also observed a potential increase in the expression levels of activation marker CD44 on CD4 and CD8 T cells in the lungs of the mice that had been injected with NCOR2 KD tumor cells, indicating that tumor cells that express NCOR2 also repress anti-tumor T cell activity (Fig. 5P, Q).

### NCOR2 permits metastatic colonization of breast cancer cells by repressing interferon gamma-mediated MHC I expression

**Fig. 6:**
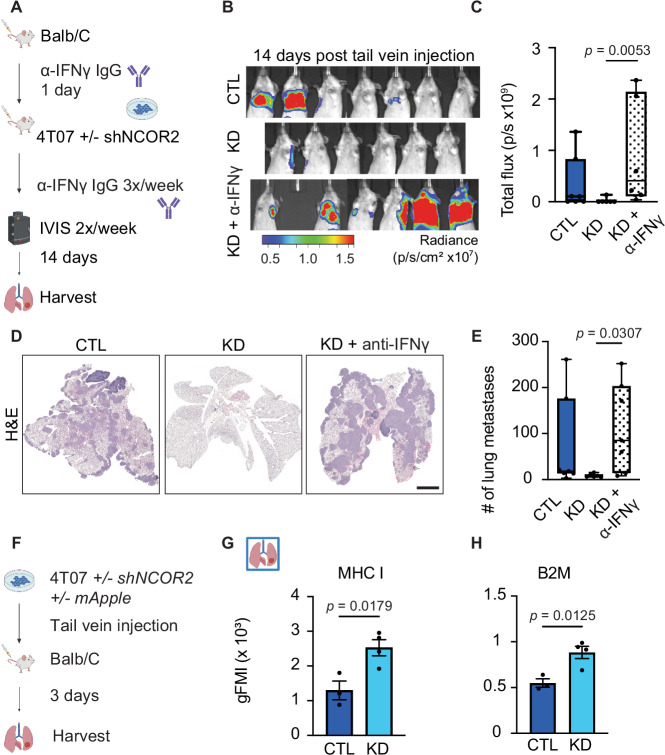
NCOR2 modulates CD8 T cell lung infiltration in 4T07 tail vein injected syngeneic mice. **A** Cartoon illustrating experimental design. **B** Representative bioluminescence images of Balb/C mice 14 days following tail vein injection with 4T07 cells with (KD, *n* = 12 total mice) or without (CTL, *n* = 7 mice) knockdown of NCOR2. Mice receiving cells with NCOR2 KD were subdivided into treatment groups with a control IgG (KD, *n* = 6 mice) or an antibody targeting the IFNγ receptor (KD + α-IFNγ, n = 6 mice). **C** Box and whisker plots showing quantification of the BLI total radiance for the mice in **B** (CTL, *n* = 7 mice; KD, *n* = 6 mice; KD + α-﻿IFNγ, *n* = 6 mice). **D** Representative images of hematoxylin and eosin (H&E)-stained FFPE tissue sections derived from the lungs of the mice in **B**. Scale bar: 1 mm. **E** Box and whisker plots showing H&E determined quantification of the number of lung metastases for the mice in **B** (CTL, *n* = 7 mice; KD, *n* = 6 mice; KD + α-﻿IFNγ, *n* = 7 mice). **F** Cartoon illustrating experimental design. **G**, **H** Bar graphs showing flow cytometry analysis of lung tissues harvested from mice injected with mApple expressing 4T07 cells with (KD, *n* = 4 mice) or without (CTL, *n* = 3 mice) knockdown of NCOR2 as in **F**, to determine the geometric mean fluorescence intensity (gMFI) of MHC-I (**G**) and β2-Microglobulin (B2M; **H**) on the surface of 4T07 cells. Box and whisker plots are represented as median (centre line) and interquartile range (box), whiskers indicate minimum to maximum values. Statistical analysis was performed using a Kruskal–Wallis test with Dunn’s multiple comparisons (**C**, **E**). Bar graphs are represented as mean ± s.e.m. using a two-sided unpaired *t* test (**G**, **H**). CTL control, KD knockdown, α-﻿IFNγ anti-interferon gamma. Graphical elements were created with BioRender.com.

To study the mechanisms by which NCOR2 modulates anti-tumor immune activity responses against metastatic breast cancer cells, we focused on the role of IFNγ. IFNγ is known to increase antigen expression via modulation of MHC-I localization to the cell surface and could thereby improve recognition by anti-tumor T cells/stimulate CD8^+^ T cell recruitment and/or activity^42,77^. To study the impact of NCOR2 through IFNγ, we repressed IFNγ signaling in the 4T07 breast cancer cells with (4T07-KD) and without (4T07-CTL) NCOR2 knockdown using a function-blocking antibody to IFNγ. We then tested the impact on lung metastasis following tail vein injection. Cohorts of syngeneic host mice that were treated with IFNγ inhibitory antibody or a matched isotype control were injected with 4T07-CTL and -KD cells via tail vein (Fig. 6A). The mice were monitored twice weekly for metastatic lung growth using BLI, and the excised lungs were examined for the number and size of the metastatic lesions at study termination. We observed a striking effect of blocking IFNγ on the metastatic lung outgrowth of 4T07 cells with an NCOR2 knockdown. NCOR2 knockdown severely impaired lung metastatic outgrowth, while mice receiving 4T07-KD cells and the anti-IFNγ IgG showed a similar image intensity as the mice injected with 4T07-CTL cells (Fig. 6B, C). Gross analysis of the lungs excised from mice clearly demonstrated a reduction in metastases (overall metastatic burden) on the surface of the lungs receiving 4T07 cells with NCOR2 knockdown, and this difference was obliterated when IFNγ signaling was prevented (Fig. 6D and Supplementary Fig. 5A). Furthermore, H&E staining of FFPE lung tissue sections revealed that although there was a striking reduction in the total number of metastases and metastatic lesion area in the lungs of mice injected with 4T07-KD cells, once again, this difference was obliterated when IFNγ signaling was inhibited (Fig. 6E and Supplementary Fig. 5B). Consistently, while the intensity of CD45 + IHC staining in the lungs from the mice in which NCOR2 was knocked down was higher than that observed in the lungs from the mice injected with 4T07-CTL cells, the levels were reduced when the tumor cells were injected with anti-IFNγ (Supplementary Fig. 5C, D). The findings implicate inhibition of IFNγ signaling as an important mechanism whereby NCOR2 permits metastatic outgrowth, and imply altered regulation of anti-tumor immunity in this phenotype.

IFNγ is known to increase antigen presentation on tumor cells by modulating the expression of molecules that regulate MHC molecule cell surface expression^78^. We therefore assayed for the impact of tumor cell NCOR2 expression on MHC class I cell surface expression. Cohorts of mice were injected via tail vein with 4T07 breast cancer cells expressing mApple that were additionally engineered to inducibly express an shRNA targeting NCOR2 or alternatively transduced with an empty vector control. Three days following tail vein injection of the modified 4T07 tumor cells, the lungs were excised and the tumor cells were subjected to flow cytometry analysis (Fig. 6F and Supplementary Fig. 5E). Flow cytometry revealed a significant increase in MHC class 1 and B2M cell surface expression on the 4T07 breast tumor cells isolated from the lungs of the mice in which NCOR2 had been knocked down (Fig. 6G, H). The findings are consistent with the higher levels of activated CD8 T cells and apoptosis detected in the tumor cells within the lungs of the mice injected with 4T07 cells in which NCOR2 was knocked down.

### NCOR2 inhibits Tap1, Tap2, and Tapbp transcription to repress MHC class I surface expression

**Fig. 7:**
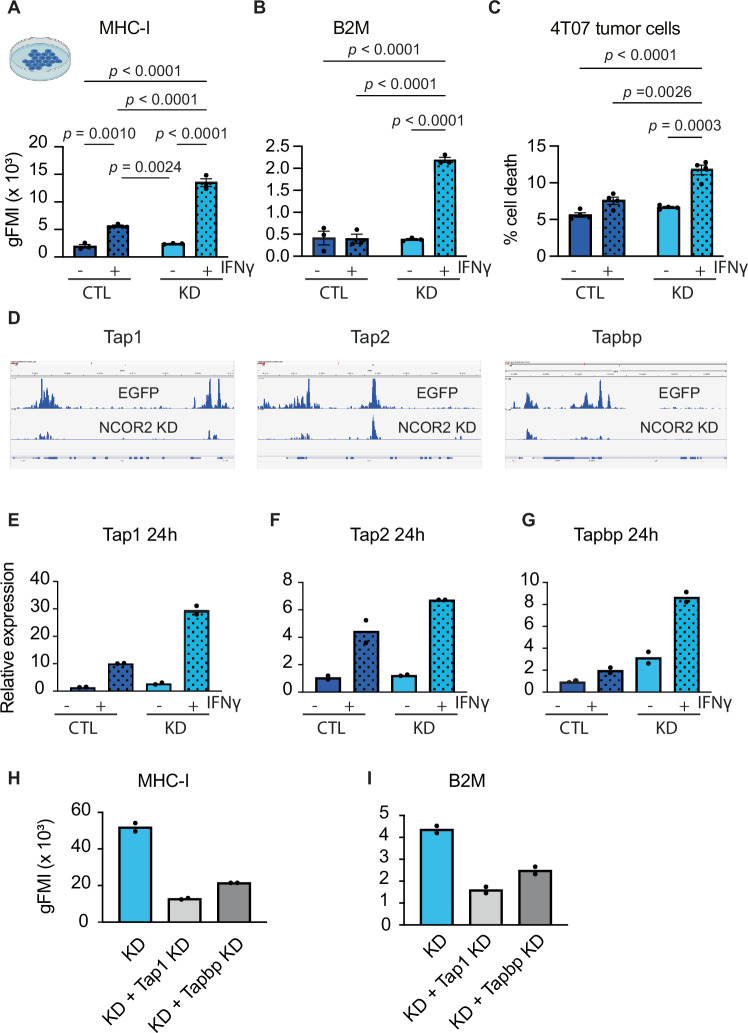
NCOR2 modulates lung metastatic outgrowth through INFγ signaling. **A**, **B** Flow cytometry analysis of cultured 4T07 cells with (KD, *n* = 3 independent experiments) or without (CTL, *n* = 3 independent experiments) knockdown of NCOR2 and stimulated +/- IFNγ for 48 h. Bar graphs show quantification of geometric mean fluorescence intensity (gMFI) of MHC-I (**A**) and β2-microglobulin (**B**, B2M) detected on the surface of 4T07 cells. **C** 4T07 cells cultured as in **A** and **B** were fixed and stained with a viability dye (Live-or-Dye) to quantify the percentage of cell death induced by IFNγ stimulation (CTL, *n* = 4 independent experiments; KD, *n* = 4 independent experiments). **D** CUT&Tag analysis illustrating peak profiles comparing genomic occupancy of NCOR2 at the loci of the *Tap1* (log2FC = 1.2, *p*-value < 0.05), *Tap2* (log2FC = 1.46, *p*-value < 0.01; log2FC = 1.5, *p*-value < 0.0001) and *Tapbp* (log2FC = 0.8, *p*-value < 0.01) genes in 4T07 cells with (NCOR2 shRNA) and without (EGFP shRNA) NCOR2 knockdown. log2FC = Log2 fold change in expression; *p*-value obtained using two-sided Wilcox Rank Sum test followed by Benjamini–Hochberg (BH) correction. Bar graphs showing quantitative RT-PCR analysis of *Tap1* (**E**), *Tap2* (**F**) and *Tapbp* (**G**) expression relative to *Gapdh* for 4T07 cells with (KD, *n* = 2 independent experiments) or without (CTL, *n* = 2 independent experiments) knockdown of NCOR2 cultured and stimulated +/- IFNγ for 24 h. **H**, **I** Flow cytometry analysis of 4T07 cells expressing NCOR2-targeting shRNA alone (KD, *n* = 2 independent experiments) or in combination with either *Tap1*- (KD + *Tap1* KD, *n* = 2 independent experiments) or *Tapbp*- (KD + *Tapbp* KD, *n* = 2 independent experiments) targeting shRNA cultured and stimulated with IFNγ for 48 h. Bar graphs show quantification of gMFI for MHC-I (**H**) and β2-microglobulin (**I**, B2M) detected on the surface of 4T07 cells. Bar graphs are represented as mean ± s.e.m. using a two-way ANOVA with Tukey’s multiple comparisons (**A**–**C**). CTL control, KD knockdown, IFNγ interferon gamma. Graphical elements were created with BioRender.com.

We next sought to clarify the molecular mechanisms whereby NCOR2 repressed antigen presentation through modulation of MHC-I cell surface expression in the breast tumor cells. We first established an in vitro system to study NCOR2-dependent MHC-I cell surface expression. We examined MHC class I and B2M cell surface expression in cultured 4T07 breast cancer cells with and without NCOR2 knockdown and IFNγ treatment. Flow cytometry studies revealed that treating cultured NCOR2 KD 4T07 and 4T07-DeCOR2 breast cancer cells with IFNγ appeared to induce higher cell levels of surface expression of MHC-I and B2M (Fig. 7A, B, Supplementary Fig. 6A, B). The findings are consitent with the observed increase in MHC class I expression detected in the 4T07 breast tumor cells that were isolated from the lungs in which NCOR2 was knocked down following their tail vein injection. Importantly, we also observed an induction of apoptosis in the cultured NCOR2 knockdown 4T07 cells in response to IFNγ treatment (Fig. 7C). The apoptosis findings are consistent with our earlier results showing elevated cell death, indicated by higher cleaved caspase 3, in the lung metastatic lesions from the spontanenous PyMT tumors in which NCOR2 was knocked out (Fig. 3I, J), as well as in the lung metastases present in the mice injected with 4T07-NCOR2 KD cells (Fig. 4F, G), or 4T07-DeCOR2 cells with pertrubed HDAC3 repressor function (Fig. 4M, N).

After establishing an in vitro model to explore how NCOR2 regulates tumor cell surface expression of MHC class I molecules, we next conducted CUT&Tag studies with NCOR2 to identify candidate genes whose gene occupancy could explain the NCOR2-mediated repression of MHC class I cell surface levels^79,80^. We generated 4T07 breast tumor cells with/without NCOR2 knockdown or EGFP scrambled vector and treated the cell lines either with vehicle or IFNγ (0 and 6 h), with at least 4–6 samples per comparison group to generate statistical robustness. MACS3 was used with filtered high-quality reads to establish peak enrichment (see “Methods” for details). A False Positive Detection Rate (FDR) of 0.01 was used for peak enrichment analysis. Extensive read quality assessment and statistics have been summarized and reported in the MultiQC file provided as supplementary data. CUT&Tag cistrome identification identified approximately 51,000 significant peaks that bound NCOR2 across all samples. We did not restrict ourselves to transcription start sites at the beginning of a gene but rather performed a completely unbiased peak enrichment analysis. Hence, many peaks were identified at the intronic regions in the middle of genes. In addition, several appearing to be single peaks were subdivided for independent analysis of the subpeaks. The peaks were distributed across all the chromosomes of the mouse genome with marginal distribution on the X chromosome and negligible distribution on the Y chromosome. There was also a sparse distribution of peaks on the mitochondrial chromosome. The raw count data for peaks in each sample have been provided as a supplementary file. Genomic distributions of the peaks for every sample, including controls, have been made available in files with the naming convention <Sample_Name > .markdup.q30_peaks.xls. Out of the ~51,000 peaks that were identified, 15,786 peaks showed a significant diminution upon NCOR2 knockdown. Out of these, only 27 of the identified peaks were marginally upregulated in NCOR2 knockdown, while the remaining were downregulated. An integrated raw counts assessment for each peak in every sample has been provided in the supplementary data section.

Unsurprisingly, consistent with prior studies, we did not identify the Tap genes as the top significant genes that were diminished following NCOR2 knockdown. Instead, the top significant genes included *Sos2*, *Adamts7*, *Vwa3b*, *Ccdc126*, as well as members of the NFκB gene regulatory circuit and a few members of the multicatalytic proteosome complex (see Supplementary Dataset 1 for a full list with statistical ordering and analysis and Supplementary Figs. showing peak binding of additional genes—Supplementary Fig. 6C–G). Therefore, we cannot rule out that MHC Class 1 molecule cell surface expression could be regulated by NCOR2 indirectly through the activity of several parallel pathways, including NFκB signaling and via altered proteolytic degradation. Nevertheless, the Tap genes did show a strong statistical significance for *Tap1* (log2FC = 1.2, *p*-value < 0.05, Rank = 15527), *Tap2* (log2FC = 1.46, *p*-value < 0.01, Rank = 8608; log2FC = 1.5, *p*-value < 0.0001, Rank = 801) and *Tapbp* (log2FC = 0.8, *p*-value < 0.01, Rank = 8011) where the fold change values represent overexpression in EGFP control vs NCOR2 knockdown (Fig. 7D). Moreover and importantly, *Tap1*, *Tap2*, and *Tapbp* have been strongly implicated in directly modulating MHC Class 1 processing. For example, MHC-I antigen processing in tumor cells depends upon the transport of endogenous degraded proteins into the endoplasmic reticulum (ER) lumen by the transporter associated with antigen processing (TAP) complex, which is composed of Tap1 and Tap2 proteins. Within the ER, MHC class I molecules are then assembled into a peptide loading complex (PLC) together with β2-microglobulin (B2M) and other proteins (tapasin, ERp57, and calreticulin), trimmed by ER enzymes, and then optimized by Tap binding protein-related protein (Tapbp). Thus, although future work will need to explore the role of other candidate NCOR2-bound genes in MHC Class 1 presentation, we opted to assess the role of NCOR2-mediated *Tap* gene regulation in MHC class I cell surface expression. Quantitative RT-PCR analysis demonstrated that *Tap1*, *Tap2*, and *Tapbp* appeared to be repressed by NCOR2 expression, as was *Nlrc5*, an MHC class I transactivator (Fig. 7E–G, and Supplementary Fig. 6H–L)^81^. Furthermore, shRNA-mediated knockdown of either Tap1 or Tapbp exhibited a trend toward prevention of the increased cell surface expression of MHC-I in the 4T07 breast tumor cells, even when coupled with NCOR2 knockdown (Fig. 7H, I). The findings identify NCOR2-mediated repression of the genes *Tap1* and *Tap2*, which regulate antigen import into the ER, and repression of *Tapbp* gene to optimize antigen assembly into the MHC class I complex before transport to the cell surface, as one plausible direct mechanism whereby NCOR2 could repress MHC class I cell surface neoantigen presentation. Our findings suggest that NCOR2-mediated MHC class 1 cell surface neoantigen presentation would thereafter compromise CD8 T cell activation and tumor cell clearance, thereby permitting metastatic outgrowth. Additional future studies will need to be conducted to further assess this possibility.

### Association between NCOR2 and MHC class I expression in human breast cancer cells, CD8 T cells, and breast cancer metastasis

**Fig. 8:**
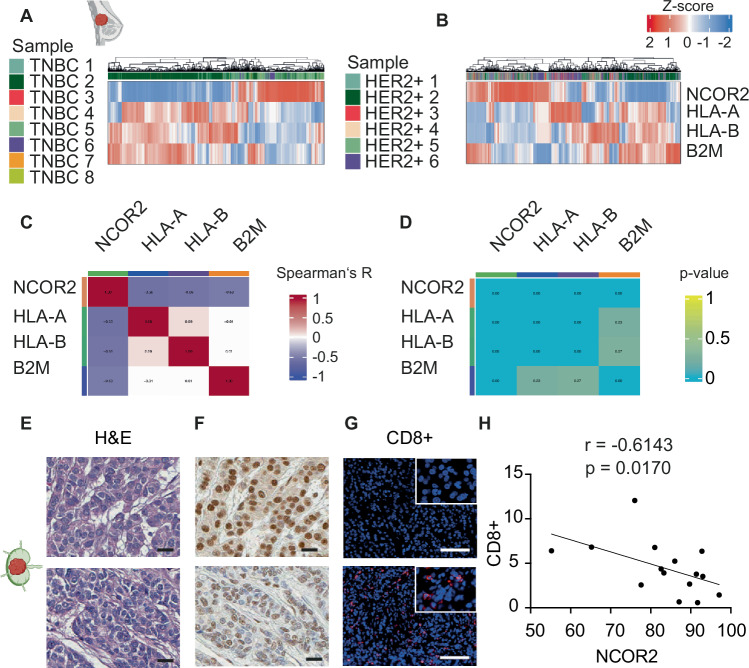
Association between NCOR2, CD8 T cells, and IFNγ-dependent gene expression in human metastatic breast cancers. Heatmaps of NCOR2 expression versus MHC class I genes from the primary tumors of breast cancer patients using publicly available single cell RNA sequencing datasets (See methods), including triple negative (TNBC patients, *n* = 8) (**A**) and HER2 positive breast cancers (HER2+ pateints, *n* = 6) (**B**). **C** Spearman correlation heatmap between NCOR2, HLA-A, HLA-B and B2M showing significant correlation between NCOR2 and MHC genes (TNBC patients, *n* = 8; HER2+ pateints, *n* = 6). **D** Two-sided *p*-value heatmap showing significance rank associations of findings shown in **C** (TNBC patients, *n* = 8; HER2+ pateints, *n* = 6). **E** Representative images of FFPE tissue sections from human breast cancer lymph node metastases stained for hematoxylin and eosin (H&E). Scale bar: 20 μm. **F** Representative images of immunohistochemical (IHC) staining of FFPE tissue sections as described in **E** using an antibody specific to NCOR2. Scale bar: 20 μm. **G** Representative images of immunofluorescence (IF) staining of FFPE tissue sections as described in **E** using an antibody specific to CD8. Scale bar: 100 μm. **H** Scatter plot showing the Spearman’s correlation between the percentages of CD8+ immune cells and NCOR2+ lymph node metastatic breast cancer cells processed as in **F** and **G** (matched primary tumor and lymph node metastasis tissues from patients, *n* = 15). The correlation coefficient (RS) and *P* values are shown for **F**. Graphical elements were created with BioRender.com.

Our murine studies implicated NCOR2 as an important regulator of the metastatic outgrowth of TNBC cells, first by thwarting innate immunity, and thereafter by repressing IFNγ signaling in the tumor cells to repress CD8 T cell activity and compromise apoptosis induction and MHC class I cell surface expression in the disseminated tumor cells. Accordingly, high expression of NCOR2 in human breast cancers should correlate with reduced MHC class I molecule expression and decreased numbers of CD8 T cells in primary and metastatic human breast tumors. Consistent with this prediction, bioinformatics analysis of 10,676 single cells from 6 primary HER2+ and 21,420 single cells from 8 TNBC human tumors that included quantification of *NCOR2*, *HLA-A*, *HLA-B*, and *B2M* mRNA, revealed that tumor cells with high NCOR2 expression display a striking inverse correlation with these MHC class I molecules (Fig. 8A, B). Heatmaps for (i) Spearman correlation values between *NCOR2*, *HLA-A*, *HLA-B* and *B2M*, and (ii) *p*-values for Spearman correlations between each pair of genes revealed a significant correlation (Fig. 8C, D). Furthermore, IHC analysis of lymph node metastatic lesions in human TNBCs revealed a significant inverse correlation between high nuclear NCOR2 expression and CD8 T cell infiltration (Fig. 8E–H). These findings provide evidence that human breast tumors expressing high levels of nuclear NCOR2 gain a competitive advantage to form progressive metastatic lesions because they exhibit compromised IFNγ signaling that represses their MHC class I molecule cell surface expression and apoptosis induction in response to T cell secreted IFNγ. Reduced MHC class I-dependent neoantigen presentation on NCOR2 high tumor cells would also likely compromise CD8 T cell activation favoring tumor cell survival and metastatic outgrowth^31^.

## Discussion

Our findings identify the nuclear corepressor protein NCOR2 as a previously unrecognized epigenetic regulator of immune escape that promotes metastatic progression in breast cancer. Our studies revealed that NCOR2 permits metastasis by repressing the expression of cytokines that recruit and activate anti-tumor immune responses at the metastatic site that normally clear disseminated tumor cells. We showed that NCOR2 further facilitates metastatic progression by reducing tumor cell IFNγ signaling and preventing the upregulation of MHC class I molecules on tumor cells to permit immune evasion. We showed that reducing tumor cell NCOR2 levels potentiates IFNγ-dependent killing, permits upregulation of class I MHC molecules in disseminated tumor cells by increasing expression of key epitope presentation genes *Tap1*, *Tap2*, and *Tapbp*, and promotes the influx of CD4/8-positive T cells, neutrophils, macrophages, and NK cells at the metastatic lung site to ultimately restrain metastatic colonization. Consistently, patients whose TNBC and HER2+ breast tumors expressed high levels of *NCOR2* also had low levels of MHC class I molecules *HLA-A*, *HLA-B*, and *B2M*, and the metastatic lesions of patients with TNBC showed a significant inverse correlation between CD8 T cell infiltration and nuclear NCOR2 expression. The findings expand the repertoire of targetable epigenetic factors that regulate antigen presentation via MHC class I on tumor cells to modulate anti-tumor immunity^82–85^. Identification of NCOR2 as an epigenetic regulator of the innate immune response at the metastatic site and of MHC class I-dependent neoantigen presentation presents an attractive anti-tumor target towards which treatments that impede its function could be designed and implemented to prevent metastatic outgrowth and enhance patient survival.

Immune checkpoint inhibitors and TCR-based therapeutics (cell therapies and engagers) have emerged as exciting approaches to eliminate and eradicate metastases^86–90^. Although immune checkpoint treatment has shown encouraging promise for some solid cancers, including groups of patients with TNBCs, there remains considerable room for improvement^91–94^. A major regulator of CD8 T cell activity and efficient antitumor immunity is MHC class I antigen presentation^34,37,38^. Not surprisingly, tumors frequently acquire a plethora of irreversible and reversible molecular mechanisms that reduce their MHC class I expression and/or neoantigen presentation to evade recognition by the immune system^39,44,95^. Our findings identify NCOR2 as an important epigenetic repressor of anti-tumor immunity through its ability to inhibit antigen assembly and cell surface presentation with MHC class I via repression of *Tap1*, *Tap2*, and *Tapbp* transcription. Because NCOR2 can also modulate NFκB transcription, the discovery expands the repertoire of reversible and targetable mechanisms that compromise IFNγ and NFκB signaling that tumors adopt to decrease MHC class I-mediated antigen presentation to evade immune detection and compromise anti-tumor immunity^46–50^. Indeed, it is also possible that NCOR2 indirectly regulates MHC Class 1 cell surface neoepitope presentation through parallel pathways, including NFκB signaling.

Mechanisms dictating dormancy versus metastatic progression include factors regulating the angiogenic switch, stress programs that control tumor cell growth versus stasis, as well as tumor stemness and tumor microenvironmental factors^96–99^. Efficient immunosurveillance is another powerful mechanism dictating dormancy, including efficient activation of an initial innate immune response as well as antigen presentation by MHC class I molecules^15,17^. We identified NCOR2 as an important promoter of metastatic progression through its ability to repress both the initial innate immune response as well as to inhibit the transcription of key molecules involved in antigen presentation via MHC class I molecules in disseminated tumor cells. The findings highlight a key role for NCOR2 in dictating early metastatic progression and suggest that factors that induce NCOR2 may play a key role in driving tumor recurrence. Consistently, NCOR2 expression is induced by Notch signaling^100,101^, and Notch has been implicated in metastatic recurrence^102,103^. Interestingly, NCOR2 also directly interacts with the dormancy-inducing transcription factor orphan hormone receptor NR2F1, which may inhibit its function to prevent tumor cell stasis and dormancy and instead promote metastatic progression^104^. Accordingly, clarifying factors regulating NCOR2 levels and function may also provide valuable insight into tumor dormancy and metastatic recurrence.

## Methods

Our research complies with all relevant ethical regulations. Our animal research studies conform to protocol #AN207099 approved by the Institutional Animal Care Use Committee (IACUC). Our human subject research studies were approved by Institutional Review Board (IRB) protocols #10-03832 and #10-05046 (UCSF) and #Pro00054515 (Duke University).

### Survival analysis

Kaplan–Meier plots were generated using the KMplot plotter (http://kmplot.com). NCOR2 expression was dichotomized using a cut-off value of 69 for distant metastasis-free survival (DMFS) in HER2-positive breast cancer, and 87 in triple-negative breast cancer (TNBC). To avoid missing correlations due to the use of a specific cutoff, all available cutoff values between the lower and upper quartiles of expression are used for the selected gene, and false discovery rate (FDR) using the Benjamini–Hochberg method is computed to correct for multiple hypothesis testing. The cutoff value with the highest significance (lowest FDR) is determined. In case of multiple cutoff values with identical significance, the cutoff with the highest hazard rate (HR) is selected for the final analysis.

### Human breast tissue acquisition and processing

Human breast tumor specimens were obtained from patients with breast cancer following informed consent from the University of California, San Francisco (UCSF) between 2010 and 2020. Tissue specimens were flash frozen in OCT (Tissue-Tek) by slow immersion in liquid nitrogen or placement on dry ice and kept at −80 °C until cryosectioning. All specimens were de-identified, stored, and analyzed according to the procedures described in IRB protocols #10-03832 and #10-05046 (UCSF) and #Pro00054515 (Duke University). Human breast cancer tissue microarrays were obtained from US Biomax (Rockville, MD, USA).

### Cell culture

4T1 and 4T07 cells were grown on tissue culture plastic in RPMI with 10% FBS and antibiotics, and HEK-293 cells were grown on tissue culture plastic in DMEM with 10% FBS and antibiotics. Growth medium was changed 1 day after plating and every 2 days thereafter. Cell lines were tested for Mycoplasma (MycoAlert Mycoplasma Detection Kit, Lonza; Cat. #LT07-318). All cell lines were derived from authenticated sources.

### Cell growth curve

4T07 cells were grown on tissue culture plastic in RPMI with 10% FBS and antibiotics. Part of the cells were pretreated with 2 μL/mL of 0.1 M IPTG for 4 days. Thereafter, the cells were seeded at a density of 7000 cells per well in a Corning 12-well plate (3.6 cm^2^, Fisher Scientific; Cat. #10253041). Counting was conducted using a hemocytometer chamber, every 24 h for 6 days.

### Lentiviral infection and vectors

Stable knockdown of NCOR2 was achieved by lentivirus-mediated RNA interference (RNAi). Sequences of validated shRNA against murine NCOR2 were obtained from the Broad Institute Genetic Perturbation Platform (GPP Web Portal - Gene Details (broadinstitute.org) and cloned into two custom built 2nd generation (shRNA) IPTG inducible vectors between AgeI and EcoRI sites using the U6 inducible 3 × LacO developed at the Broad Institute (Vector Details: pLI_913 (broadinstitute.org). Murine NCOR2 shRNA included TRCN0000238139 (TGTTGGCTTACAGGGTATATT) validated in this study, and TRCN000095283 (CCAGATCATCTACGATGAGAA), and TRCN000095281 (CCAGTGTAAGAACTTCTACTT) validated by Sigma Mission. An shRNA targeting EGFP was used as non-specific control (GCAAGCTGACCCTGAAGTTCAT). 4T1 and 4T07 cells transduced with these constructs were selected with up to 1 mg/ml G418 and hairpins induced with 200 µM IPTG (in vitro) or 20 mM Dioxane Free IPTG in drinking water provided ad libidum (in vivo). Cells were FACS-sorted for mApple expression. Infection was verified by immunoblot analysis. The luciferase expression was achieved using a lentivirus-mediated mApple and firefly luciferase (FF-Luc) fusion vector. Stable knockdown of *Tap1* and *Tapbp* was achieved by lentivirus-mediated RNAi. Murine shRNA sequences were obtained as bacterial glycerol stocks from Sigma-Aldrich (www.sigmaaldrich.com), harboring lentiviral plasmids cloned into the pLKO.1-puro vector. The shRNA sequence targeting *Tap1* was TRCN0000066349 (CGCATCACTGACTGGATTCTT), validated in-house by qPCR, and for *Tapbp* was TRCN0000112632 (CCCAAAGCTATACTTCAAGGT), previously validated by Sigma Mission. Bacterial cultures were expanded from these stocks, and plasmid DNA was purified using standard maxiprep protocols. Lentiviral particles were produced in HEK293T packaging cells via co-transfection with packaging plasmids (psPAX2 and pMD2.G), and collected supernatants were used to transduce target 4T07 CTL and shNCOR2 expressing cell lines. Transduced cells were selected with puromycin (2 µg/ml), and knockdown was confirmed by qPCR. The construction of the tet-regulated expression vector for Myc-tagged mDeCOR2, using a PiggBac transposon system is based on what has been previously described, with the exception that this vector was also engineered to express both the luciferase and reverse tet Transactivator (rtTA) genes^53^. Cells transduced with this vector were selected using neomycin.

### Transgenic experiments

All animal studies involved female mice. Animal numbers are indicated in the figure legends. NCOR2 knockout mice (PyMT MMTV-Cre NCOR2 KO) were generated by crossing MMTV-PyMT with MMTV-Cre NCOR2 KO mice^105^. All strains were on an FVB/NJ background (Strain# 001800). Animal husbandry and procedures were conducted in UCSF Parnassus Laboratory Animal Resource Center (LARC) facilities in compliance with protocol #AN207099 approved by the Institutional Animal Care Use Committee (IACUC). Once a week, tumors were measured using calipers. Mice were sacrificed at 13–22 weeks once a single tumor reached 1.5 cm in diameter. Mice were anesthetized with 5% inhalant isoflurane. Then, under anesthesia, retroorbital blood was collected from the venous sinus, and a transcardial perfusion was performed by opening the thoracic cavity. A blunt-tip needle was inserted into the right ventricle, and a small incision was made in the left atrium. Transcardial perfusion was performed with 30–40 mL PBS. While still attached in situ, lungs were directly perfused via the trachea with 1 mL of 4% formaldehyde to avoid collapsing. The lungs were dissected, surface metastases were macroscopically calculated, and the tissues were paraffin-embedded for subsequent analysis. Mammary tumors from euthanized mice were harvested and paraffin-embedded, cryo-embedded, and snap-frozen tumor chunks were collected.

### Tail vein experiments

For tail vein experiments, 4T07 cells with or without NCOR2 knockdown and with or without DeCOR2 expression and luciferase expression were used. Four days before the injection, cells were pretreated with 2 μL/mL of 0.1 M IPTG. Female Balb/C (Strain #000651) and NSG (NOD.Cg-Prkdc^scid^ Il2rg^tm1Wjl^/SzJ, Strain #005557) mice were obtained from The Jackson Laboratory, and animal numbers are indicated in the figure legends. Animal husbandry for mice was carried out in the LARC facilities at UCSF. 8-week-old female Balb/C mice were administered IPTG in the drinking water at 5 mg/mL. The following day, mice were injected with 10^6^ cells suspended in 100 μl of PBS into the tail vein. Lung metastases were monitored biweekly by bioluminescence imaging. Administration of IPTG continued until sacrifice at 3, 7, and 14 days following tail vein injection. Twenty-nine-week-old female NSG mice were administered IPTG water at 5 mg/mL. The following day, mice were injected with 10^6^ cells suspended in 100 μl of PBS into the tail vein. Lung metastases were monitored biweekly by bioluminescence imaging. Administration of IPTG continued until sacrifice at 10 days following tail vein injection. 9-week-old female Balb/C mice were administered IPTG in the drinking water at 5 mg/mL. Then, mice were divided into three groups. The first group was injected intraperitoneally with 500 μg control IgG (Rat IgG1 Isotype Control, Leinco Technologies; Cat. #R1379) diluted in PBS; the following day, mice were injected with 10^6^ 4T07 control cells suspended in 100 μl of PBS into the tail vein. The second group was injected intraperitoneally with 500 μg control IgG (Rat IgG1 Isotype Control, Leinco Technologies; Cat. #R1379) diluted in PBS; the following day, mice were injected with 10^6^ 4T07 NCOR2 knockdown cells suspended in 100 μl of PBS into the tail vein. The third group was injected intraperitoneally with 500 μg anti-IFNγ (anti-Mouse IFNγ, Leinco Technologies; Cat. #I-1209) diluted in PBS; the following day, mice were injected with 10^6^ 4T07 NCOR2 knockdown cells suspended in 100 μl of PBS into the tail vein. Lung metastases were monitored biweekly by bioluminescence imaging. Administration of IPTG continued until sacrifice at 14 days following tail vein injection. At the end of the experiment, lung perfusion and animal euthanasia were performed as described above, and lungs were paraffin-embedded. For flow cytometry analyzes, lungs were perfused only with PBS and stored in a mixture of 95% FBS and 5% DMSO at −80 °C.

### Orthotopic experiments

For orthotopic experiments, 4T1 cells with or without NCOR2 knockdown were used. Cells were pretreated with 2 μL/mL of 0.1 IPTG 4 days before the injection. Female Balb/C mice were obtained from The Jackson Laboratory, and numbers are indicated in the figure legends. Animal husbandry for mice was carried out in the Laboratory Animal Resource Center (LARC) facilities at UCSF. 16-week-old female Balb/C mice were administered IPTG drinking water at 5 mg/mL. The following day, mice were anesthetized using 3.5% inhalant isoflurane, and Buprenorphine and Meloxicam intraperitoneal anesthesia, and ophthalmic ointment was applied. Hair over the area surrounding the left 4th mammary gland was removed, and the skin was wiped using 70% alcohol, followed by 2% chlorhexidine solution. Then, a 0.5 cm incision between the 4th nipple and the midline was made, and the skin was gently separated from the underlying fascia using a cotton swab and blunt dissection. The 4th fat pad was located and stabilized using micro-dissecting forceps and injected with a mixture of 10^5^ 4T1 cells with or without NCOR2 knockdown, suspended in 50 μl of PBS and 50 μl of Matrigel. Then, the incision was closed, and a topical local analgesic (Lidocaine) was applied. Postsurgical intraperitoneal anesthesia with Buprenorphine and Meloxicam was performed. Tumors were measured using a caliper biweekly. Administration of IPTG continued until sacrifice at 4 weeks following mammary fat pad injection. At the end of the experiment, retroorbital blood collection, lung perfusion, and animal euthanasia were performed as described above. The lungs were dissected, surface metastases were macroscopically calculated, and the tissues were paraffin-embedded for subsequent analysis. Mammary tumors from euthanized mice were harvested and paraffin-embedded, cryo-embedded, and snap-frozen tumor chunks were collected.

### Human TNBC xenograft model

PDX tissues were obtained from Dr. Alana Welm at the Huntsman Cancer Institute, University of Utah, Utah. Equal-sized pieces of human triple-negative PDX BCM2665 were inserted into the mammary glands of female 8-week-old NOD SCID (NOD.Cg-Prkdc^scid^/J, Strain #:001303) mice in soft or ribose-stiffened collagen gels. Mice were obtained from The Jackson Laboratory, and numbers are indicated in the figure legends. Animal husbandry for mice was carried out in the LARC facilities at UCSF. Paclitaxel injections (10 mg/kg) began twice weekly, 3.5 weeks after PDX implantation, following the emergence of tumors with an average size of 9 mm. Mice were sacrificed 21 days following the start of treatment.

### Bioluminescence imaging

Bioluminescence imaging was performed using the IVIS Imaging System (Xenogen; Caliper Life Sciences) within the IACUC animal barrier at UCSF. Mice were anesthetized with 2% isoflurane and injected intraperitoneally with 150 mg/kg D-luciferin 10 min before image acquisition. Lung metastatic tissue was determined by measuring the photon flux from a region of interest drawn around the bioluminescence signal using the Live Imaging Software v.4.5.2 (PerkinElmer).

### Histology, image acquisition, and analysis

Prior to staining, paraffin-embedded tissue sections were dewaxed and hydrated through a descending grade ethanol series. Then, the sections were stained with Mayer’s hematoxylin solution (Sigma Aldrich; Cat. #MHS32) for 8 min and with Eosin Y solution (Sigma Aldrich; Cat. #E4009) for 30 s. The tissue sections were mounted with Cytoseal (Richard Allen Scientific; Cat. #48212-196). Images were acquired digitally using Leica Biosystems Microscope Slide Scanner Aperio AT2 and visualized using ImageScope software. Quantification of the number of lung metastases was performed manually. To score the percentage of metastasis area within the whole lung, the percentage of metastatic area per total lung area was calculated using QuPath.

### Immunohistochemistry, image acquisition and analysis

Prior to staining, paraffin-embedded tissue sections were dewaxed and hydrated through a descending grade ethanol series. After microwave treatment for 10 min in tris-based antigen unmasking solution (Vector Laboratories; Cat. #H-3301-250), endogenous peroxidase activity was blocked with 3% H_2_O_2_ for 10 min. Tissue sections were then blocked with 5% bovine serum albumin and 5% goat serum in PBS for 1 h at room temperature. Sections were then incubated overnight at 4 °C with primary antibodies specific to CD8a, CD45, cleaved caspase-3, Ki-67, and NCOR2. All antibodies used in this study are listed in Supplementary Dataset 2. Thereafter, sections were incubated with Impress anti-rabbit or anti-rat reagent (Novus Biologicals; Cat. #MP-5401-NB; Cat. #MP-7404-50) for 30 min at room temperature. For diaminobenzidine (DAB) staining, a positive signal was developed with ImmPACT DAB reagent (Vector Laboratories; Cat. #SK-4105). Sections were counterstained with hematoxylin and mounted with Cytoseal. Alternatively, tyramide signal amplification (TSA) was applied instead of DAB staining: sections were incubated with Alexa Fluor (Thermo Fisher Scientific; Cat. #A-11008, 1:500) diluted in Tyramide Amplification Buffer (Biotium; Cat. #33001) for 10 min. Cell nuclei were then counterstained with DAPI, sections were mounted with Vectashield, and imaged. Images of DAB-stained sections were acquired using an Olympus IX81 microscope at 40× magnification with a 0.6 Ph2 objective. Full-slide images were acquired digitally using Leica Biosystems Microscope Slide Scanner Aperio AT2 and visualized using ImageScope software. Positive staining was quantified by manually counting the nuclei of interest and total nuclei, then averaging, or using QuPath. At least five images of each tissue specimen were used for averaged analyzes.

Images of TSA-stained sections were acquired using a Nikon Eclipse TE2000-U inverted microscope at 20× magnification with a 0.75 NA objective. Images were acquired with a Hamamatsu ORCAFlash4.0 LT camera using NIS-Elements software. Full-slide images were acquired digitally using Zeiss Axio Scan Z.1. Quantification of tissue sections was performed by thresholding images at a fixed intensity and counting the percentage of positive cell nuclei per total nuclei using Fiji. 5 images of each tissue specimen were used for averaged analyses.

### Immunofluorescence, image acquisition and analysis

Frozen OCT tissue 10 μm sections were fixed in 4% PFA for 10 min at RT, and then permeabilized with 0.25% v/v Triton-X-100 in PBS for 5 min at room temperature. Tissue sections were then blocked with 5% bovine serum albumin and 5% goat serum in PBS for 1 h at room temperature. Primary antibody specific to NCOR2 was diluted in 5% bovine serum in PBS and incubated overnight at 4 °C. Tissue sections were then stained with fluorescently labeled secondary antibodies (see Supplementary Dataset 2) diluted in 5% bovine serum albumin in PBS for 1 h at room temperature. Tissue sections were counterstained with DAPI, mounted with Vectashield, and imaged. Images of IF-stained sections were acquired using a Nikon Eclipse TE2000-U inverted microscope at 20× magnification with a 0.75 NA objective. Images were acquired with a Hamamatsu ORCAFlash4.0 LT camera using NIS-Elements software. Quantification of tissue sections was performed by thresholding images at a fixed intensity and counting the percentage of positive cell nuclei per total nuclei using Fiji. Ten images of each tissue specimen were used for averaged analyses.

### Flow cytometry

Mouse lung tissue was perfused with Dulbecco’s modified PBS and either freshly chopped using a razor blade or flash frozen and chopped after rapid thawing. Chopped tissue was digested in 100 µg ml^−1^ collagenase type 1 (Worthington Biochemical; Cat. #LS004194), 500 µg ml^−1^ collagenase type 4 (Worthington Biochemical; Cat. #LS004186), and 200 µg ml^−1^ DNase I (Roche; Cat. #03724778103) while shaking at 37 °C. Digested tissue was filtered using a 100 µm filter to remove remaining pieces. Red blood cells were lysed in ACK buffer (Thermo Fisher Scientific; Cat. #A1049201), and the remaining cells were counted. Cells were stained with fluorophore-conjugated primary antibodies for 30 min on ice and subsequently stained with a viability marker. Antibodies used are listed in Supplementary Dataset 2.

### Flow cytometry for cell death, MHC class I and β2-microglobulin cell surface expression

4T07 cells expressing IPTG-inducible NCOR2-targeting shRNAs or GFP-targeting shRNA were cultured as described and treated with IPTG for 72 h prior to the reapplication of IPTG (1 mM) and stimulation with and without IFNγ for 48 h. For quantification of cell death, cell media was collected, and attached cells were harvested by trypsinization for staining with the Live-or-Dye NucFix™ Red Staining Kit (Biotium; Cat. #32010-T) for 30 min prior to two washes with PBS and fixation in 2% paraformaldehyde (PFA). For assays examining cell surface expression of MHC class I molecules and β2-microglobulin, cells were treated in the same manner as above, but also co-treated with the caspase inhibitors Ac-DEVD-CHO and Ac-IETD-CHO for 24 h prior to and throughout the duration of the experiment to avoid loss of cells due to apoptosis. Following treatment, cell media was collected, and attached cells were harvested by trypsinization for blocking in PBS + 2% Fetal bovine serum (FBS; FACS wash buffer), mouse serum and Fc receptor blocking antibody, followed by staining with antibodies against H-2Kd/H-2Dd (MHC Class I) and β2-microglobulin for an additional 30 min (for details on antibodies used see Supplementary Dataset 2). DAPI (4’,6-diamidino-2-phenylindole) was then added for 10 min to distinguish live/dead cells before washing cells with a large volume of FACS wash buffer and fixing cells in 2% PFA. Flow cytometry was performed using a BD LSRFortessa™ Cell Analyzer, and population percentages were defined and quantified using FlowJo™ v10.8 Software (BD Life Sciences).

### RNA extraction and RT-qPCR

Total RNA was extracted from the cell lysates and blood and purified using the RNeasy Mini Kit (QIAGEN; Cat. #74104). Total RNA (1.0 μg) was used as a template for cDNA synthesis using M-MLV reverse transcriptase (Promega Corporation; Cat. #M1701). cDNA (100 ng) was used as template for PCR amplification using the LightCycler FastStart DNA MasterPLUS SYBR Green I Kit (Roche; Cat. #03515869001) and the LightCycler System (Roche). Oligonucleotide primers were designed using the LightCycler Probe Design Software v.2.0 (Roche) or sourced from online datbases and all qPCR primers used in this study are listed in Supplementary Table 1. Transcript expression was quantified by normalizing the gene of interest copy number (per μl) to absolute levels of an endogenous, stably expressed reference gene HPRT. Analysis of the data was performed using the 2^-ΔΔ*CT*^ method described previously^106,107^.

### Western blotting

Protein lysates were prepared using radioimmunoprecipitation assay (RIPA; 50 mM Tris-HCl, 150 mM NaCl, 0.25% Na-deoxycholate, 0.1% SDS, and 1.0% IGEPAL CA-630 (NP-40); pH 7.4 at room temperature) or Laemmli lysis buffer (50 mM Tris-HCl, 2% SDS, and five mM EDTA; pH 7.4 at room temperature). Immediately before cell lysis, a cocktail of protease inhibitors (1.2 μg leupeptin, 1.2 μg pepstatin, 2.4 μg aprotinin, 12 μg E-64, 0.5 mM benzamidine, 50 mM NaF, and 1.2 μg Pefabloc) and the phosphatase inhibitor Na-orthovanadate (1 mM activated with 1.5% H2O2) was added to the buffer. RIPA lysates were carried out on ice, while Laemmli lysates were performed at room temperature due to SDS precipitation at cold temperatures. After rinsing the dishes with PBS, lysis buffer was added, and cells were scraped off the dish with a cell scraper. RIPA lysates were sonicated three times, each for 10 s, while Laemmli lysates were passed through a fine pipette tip (p200) several times. Both were centrifuged for 10 min at 20,817 relative centrifugal force to pellet any internal organelles and cellular debris. Supernatant was collected and fast frozen on dry ice. Protein concentration was determined using a bicinchoninic acid assay kit (Sigma Aldrich; Cat. #BCA1-KT). Protein extracts were obtained with a RIPA lysis and extraction buffer using the manufacturer’s instructions (Thermo Fisher Scientific; Cat. #89901). Equal amounts of protein were separated on reducing SDS-PA gels, immuno-blotted, and detected with the ECL Plus system (Thermo Fischer; Cat. #32132). Samples were boiled for 5 min (95 °C) and loaded onto the SDS-PA gel, and protein was separated at 120 constant volts. The protein was transferred onto a prewet polyvinylidene difluoride membrane (100% methanol, 1 min) at 300 mA for 2 h. The polyvinylidene difluoride membrane was rinsed with Tris-buffered saline with Tween 20 (TBST), and nonspecific binding was blocked with 5% nonfat dry milk dissolved in TBST. The membrane was then incubated with the primary antibody (see Supplementary Dataset 2) overnight at 4 °C, washed with TBST, incubated with HRP-conjugated secondary antibody (1 h; room temperature; dilution, 1:2000), washed with TBST, and detected with the chemiluminescence system Quantum HRP substrate (Advansta; Cat. #490005-004). Signal quantification and normalization to loading control were performed using Fiji.

### Single-cell RNAseq analyses of primary breast cancer tumors

We downloaded the single-cell RNAseq data of primary^108^ breast cancer tumors from Gene Expression Omnibus (https://www.ncbi.nlm.nih.gov/ geo/) using series GSE161529. The Seurat pipeline was applied to each sample^109^. Raw counts of annotated cancer cells were normalized (Seurat NormalizeData function) and scaled (ScaleData function). The expression of selected genes was then visualized in heatmaps using the R package ComplexHeatmap^110^.

### CUT&Tag for NCOR2 binding sites

NCOR2 binding sites were profiled using CUT&Tag as described^79^ with some modifications. Rather than using cells bound to Concanavalin A coated magnetic beads a novel format was adopted that more easily facilitated the multiple washing and incubation steps of adherent unfixed cells in situ which otherwise were easily detached and lost during the procedure. Briefly, an 11 mm (Outer Diameter) by 8 mm (Inner Diameter) gasket was punched out of 0.02” thick soft elastomer film (Durometer 35A McMaster Carr; Cat. #86435k45) and was then autoclaved and placed at the bottom of the well of a standard 48-well tissue culture plate. 9.5 mm (Inner Diameter) cell plating guide was fashioned by cutting a 1.5 cm length of the body of a 5 ml polypropylene culture tube (VWR; Cat. #60818-500) and inserted into the well. 20,000 4T07 cells from IPTG-treated NCOR2 or EGFP control knockdown cells were plated per well inside the plating guide and allowed to plate, adhere and grow in the small well produced by the gasket for 24–48 h before starting the CUT&Tag protocol. Before beginning, cells were cooled by placing plates 5 min in an ice water slurry. While on ice the plating guide insert was gently removed leaving the gasket in place. Media was aspirated, and cells were washed twice for 5 min each with 0.2 ml per well ice cold 20 mM HEPES-NaOH pH 7.5/150 mM NaCl/0.1% BSA. Before removing the last wash a 10 mm diameter disc punched from 70 µm polyester mesh (Component Supply Co. Cat. #U-CMY-70-B) was wetted and carefully placed in the well and sealed against the gasket and over the cells by another insert prepared from screw cap 2 mL tubes (Thermo Scientific Cat. #3468) repurposing the provided cap O-ring to form a liquid tight incubation chamber in which cells were isolated from washing shear forces in an ~10 µL well beneath the mesh, but yet easily able to exchange buffers and reagents through the same. To compensate for an assumed effect on bulk liquid flow mixing forces by the mesh at the cells, both incubation times with antibodies and pA-TN5 complex as well as washes were extended from those given by the original CUT&Tag method.

After forming the incubation chamber, the wash was aspirated and cells were permeabilized with 0.2 ml of ice-cold DIG buffer (20 mM HEPES-NaOH pH 7.5/150 mM NaCl/0.1% BSA/0.05% (w/v) Digoxigenin/0.5 mM Spermidine/2 mM EDTA/1× cOmplete Protease Inhibitor (Roche Cat. #11697498001)) for 15 min on ice. DIG buffer was aspirated and replaced with 50 µl DIG buffer with NCOR2 antibody (see Supplementary Dataset 2) using a lot that was tested to immunostain 4T07 nuclei. Antibody incubation was allowed to proceed overnight at 4 °C. The next day, the plate was transferred to an ice water slurry and cells were washed without agitation four times for 15 min each with 0.2 ml of ice-cold DIG buffer then incubated again overnight at 4 °C with 50 µl DIG buffer with a 1:200 dilution (5 µg/ml) of guinea pig anti rabbit IgG (Novus Cat. # NBP1-727863). Again the next day the plate was transferred to an ice water slurry and cells were washed without agitation four times for 15 min each with 0.2 ml of ice-cold DIG buffer then one time for 15 min with 0.2 ml of DIG 300 buffer (20 mM HEPES-NaOH pH 7.5/300 mM NaCl/0.1% BSA/0.01% (w/v) Digoxigenin/0.5 mM Spermidine/0.25x cOmplete Protease Inhibitor) before adding 50 µl DIG 300 buffer containing 1:250 dilution of 5.5 µM pA-TN5 prepared and loaded with annealed TN5MEDSA and TN5MEDSB oligos as described^79^. Incubation was for 2 h at room temperature while shaking on a microplate oscillator at 5–10 × *g*. Unbound pA TN5 was removed by washing four times each for 15 min at room temperature with 0.2 ml DIG 300 Buffer with shaking at 5–10 × *g*. Tagmentation was initiated by adding 50 µL DIG 300 supplemented with 12 mM MgCl and incubating for 2 h at 37°C in bacterial shaker set at 5x g. Tagmentation was stopped by adding 20 µL 4x stop buffer (20 mM HEPES-NaOH pH 7.5/300 mM NaCl/0.1% BSA/60 mM EDTA/0.4% SDS/0.6 mg/ml Proteinase K) mixing 15 min at 37 °C in bacterial shaker before floating the plate in a water bath at 55 °C for 1 h. Samples still in the plate were frozen on dry ice and stored at −80 °C before purification of tagmented DNA. Plates containing tagmented DNA were thawed, and DNA was purified by binding and elution from diatomaceous earth (DE). Briefly, to each well containing approximately 80 µL stopped tagmentation reaction was added 92 µL TE (10 mM Tris-HCl, 1 mM EDTA pH 8), 208 µL 6 M Guanidine thiocyanate (final ~2.5 M) and 50 µL 2 M (in acetate-acetic acid) K acetate – acetic acid pH 5 (final ~ 0.2 M). The plate was shaken for 15 min at room temperature at 5-10x g on a microplate oscillator to mix before well contents were transferred to a 1.5 ml microcentrifuge tube containing 20 µL of a resuspended 1:1 slurry of DE (Sigma Cat. #3877, extracted in 1 M HCl 75 °C for 4 days, washed 10× in 5–10 volumes of high purity water with 30 min settling under gravity to remove fines and suspended in 20% v/v ethanol). After a brief vortex, 50 µL of ethanol was added (final 10%), and DE was resuspended and mixed on a rotator for 5–10 min at room temperature. After pelleting DE at low speed, all but 0.2 ml of supernatant was discarded and DE resuspended and transferred to a mini column (Promega Cat. #A7211) in a 2 ml collection tube. Low-speed centrifugation was used to remove residual supernatant and facilitate washing successively with 100 µL 3 M GuHCl/50 mM Tris HCl pH 7.4/10 mM EDTA, 100 µL 50 mM Tris HCl pH 7.4/5 mM EDTA/70% Ethanol, and finally 4 successive 100 µL washes with 80% (v/v) isopropanol. DE was dried by centrifugation for 2 min at 7500 × *g* and DNA was eluted by applying 18 to 20 µL 1 mM Tris-HCl pH 8–8.5/5 mM NaCl prewarmed (~50 °C) to DE and collecting into a clean microcentrifuge tube by centrifugation for 2 min at 7500 × *g*.

### Preparation and sequencing of NCOR2 CUT&Tag libraries

16.65 µL of eluted tagmented DNA was amplified on a thermocycler in a 25 µL reaction containing 1× Phusion GC Buffer (New England Biolabs Cat. # F-519), 0.2 mM dNTP, 0.5 µM Dual 8 bp Indexed primers Ad1.# and Ad2.#^80^, and 0.35 µL Phusion Polymerase (QB3 MacroLab, UC Berkeley) using the following protocol: 1 cycle 72 °C 5 min, 1 cycle 98 °C 30 s, 14 cycles of 98 °C 15 s, 60 °C 15 s, 72 °C 1 min and 1 cycle 72 °C 2 min. A 1/100 dilution of each reaction was used to quantify libraries using qPCR with Illumina P5 (aatgatacggcgaccaccga) and P7 (caagcagaagacggcatacga) primers and a known P5/P7 amplifiable (~250 bp insert) standard run in parallel. This qPCR was repeated using normalized inputs of the same 1/100 dilution and amplification with P5 and P7 terminated at a number of cycles approximately 70–80% of maximum yield and reactions were run on ethidium bromide stained 2% agarose TAE gels to estimate library size distributions. A visual estimate of the average size of the library distribution was used as a rough (relative molar) normalization for pooling of libraries for sequencing. Pooling was performed directly with unpurified amplified libraries. The pooled libraries were then purified together and concentrated using DE as for tagmented DNA and eluted in 25 µL 1× TE for submission to the UCSF Center for Advanced Technology. Sequencing was performed on a NovaSeq X using Nextera for Illumina sequencing and index read primers with 51 bp of sequence acquired from each insert end.

### Analysis of CUT&Taq sequencing libraries

Paired reads from raw sequencing data were trimmed for Nextera adapter sequences and low-quality bases with Phred scores less than 20 using Skewer. The trimmed reads were aligned to the Mouse (Mus Musculus): mm39 build reference genome using bowtie2 and alignments with mapping quality <30 were discarded. Only the alignments with both mates of a paired-end read aligning in the right orientation were considered for peak calling. Deduplicated BAM files were generated using samtools. MACS3 was used for peak calling with background removed using IgG controls and maximum False Discovery Rate (FDR) of 1%. The peaks were called without deduplication to benefit from PCR pileup, especially for hard-to-detect peaks. Peaks were visualized as bigWig track records that were generated for each sample using bamCoverage tool from deepTools package. To visualize these tracks, duplicates were first removed by using the flag (--ignoreDuplicates) followed by normalization with the total aligned read pairs for each sample. Reads under each called peak were counted to prepare a count matrix for all consensus peaks. DESeq2 was used to perform differential expression analysis of the peaks, using the aligned reads per sample to generate scaling factor, and hypothesis testing was performed with Wilcox Rank Sum test and Benjamini– Hochberg correction used to account for multiple hypothesis testing. NCOR2 knocdown vs EGFP scrambled (control) comparisons were performed for 0 h (untreated) and 6 h timepoints (time in hours post addition of IFNγ in case of treatment and vehicle in case of untreated) to identify differentially expressed peaks. Fisher’s combined probability test^111,112^ was used to obtain the joint *p*-value of the differentially expressed peaks in 0 h and 6 h comparisons and joint log fold change expressions were obtained by averaging the individual log fold changes. Peaks with *p*-adjusted value < 0.05 were considered significant. The peaks were assigned genes by using AnnotationDbi package and implementing a criteria that assigned a peak to a gene if the peak either lies on the gene or 2 kilobases (kb) upstream of the gene.

### Statistical analysis

GraphPad Prism Version 10.4.2 was used to perform all statistical analyses and correlations, with statistical significance determined using the appropriate tests noted in the corresponding figure legends. Tests of normality, incorporating skewness and kurtosis, were employed to determine the appropriate statistical test. When the number of observations was too small to perform a formal normality test, approximate normality was assessed using visual inspection (Q-Q plot) and descriptive statistics (skewness and kurtosis). All independent variables are described in the figure legends, with measurements derived from distinct samples (biological replicates) unless otherwise stated. For all analyses, samples were randomized. Patient and mouse samples were monitored by unique identifiers, and mouse littermates were evenly distributed across different experimental groups to avoid any potential bias.

Sample sizes in mouse studies were determined based on previously published data demonstrating statistically significant differences in tumor progression and immune response using orthotopic tumor xenografts, syngeneic mouse models established with transformed mammary cells genetically engineered to express short hairpin RNA and DeCOR2, and transgenic mouse models expressing MMTV-PyMT^53,113,114^. Sample sizes for evaluating clinical studies were determined based on statistically significant differences in factors impacting tumor aggressiveness, i.e., neoadjuvant chemotherapy, as well as on sample availability^53,115^. Identification and statistical analysis of the differentially expressed peaks in the CUT & Tag experiment were performed using DESeq2 package in R 4.4.0. The combined *p*-value after multiple hypothesis correction was obtained from a total of 6 NCOR2 knockdown samples and 4 EGFP scrambled vectors as a control. A total of 5 background control samples (IgG control) were used to model the background during peak calling with MACS3.

### Reporting summary

Further information on research design is available in the Nature Portfolio Reporting Summary linked to this article.

## Supplementary information

Supplementary Information

Description of Additional Supplementary Files

Supplementary Dataset 1

Supplementary Dataset 2

Reporting Summary

Transparent Peer Review file

## Source data

Source Data

## Data Availability

The CUT&Tag data generated in this study have been deposited in the Gene Expression Omnibus (GEO) database under accession code GSE320158 (https://www.ncbi.nlm.nih.gov/geo/query/acc.cgi). The supplementary and source data generated in this study are provided in the Supplementary Information/Source Data files. The single cell RNA sequencing data used in this study are available in the GEO database under accession code GSE161529 [https://www.ncbi.nlm.nih.gov/geo/query/acc.cgi]. Source data are provided with this paper.

## Code availability

The CUT&Tag code generated and used in this study have been deposited at https://github.com/eshenderov/CUTNTag and the R script used for the analysis of single cell datasets is available at https://github.com/eshenderov/Breast-Single-Cell-Analysis.
