# Supplementary material for: NCOR2 represses MHC class I molecule expression to drive metastatic progression of breast cancer
Source: Nat Commun. 2026 May 5;17:6067. doi: 10.1038/s41467-026-72168-3 (PMC13351013; doi:10.1038/s41467-026-72168-3)
Supplement: Supplementary file 1 — Supplementary Information [file 41467_2026_72168_MOESM1_ESM.pdf]

Supplementary Information

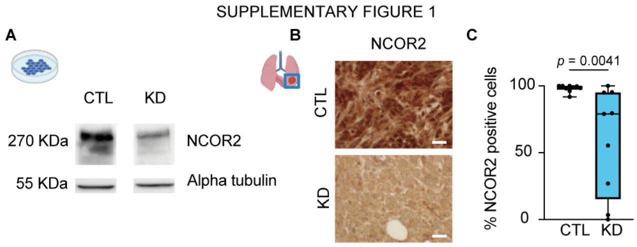

Supplementary Figure 1. Establishing an NCOR2 knockdown in 4T1 mouse mammary tumor cells. (A) Representative immunoblots using antibodies specific to NCOR2 and alpha-tubulin illustrating shRNA-mediated knockdown of NCOR2 expression in 4T1 cells. Alpha-tubulin was used as a loading control (representative data from n = 3 independent experiments). (**B**) Representative images of immunohistochemical (IHC) staining of formalin fixed paraffin embedded (FFPE) lung tissue sections with an antibody specific to NCOR2. Lung tissues contain metastatic lesions derived from 4T1 primary tumors expressing a scrambled non-targeting shRNA control (CTL) or an NCOR2-targeting shRNA (KD). Scale bar: 20 µm. (**C**) Box and whisker plots showing quantification of positive NCOR2 staining for mouse lung tissues from (B) (CTL, n = 9 mice; KD, n = 9 mice). Box and whisker plots are represented as median (centre line) and interquartile range (box), whiskers indicate minimum to maximum values. Statistical analysis was performed using a two-sided Mann-Whitney U test (C). Abbreviations: CTL, control; KD, knockdown. Graphical elements were created with BioRender.com.

SUPPLEMENTARY FIGURE 2

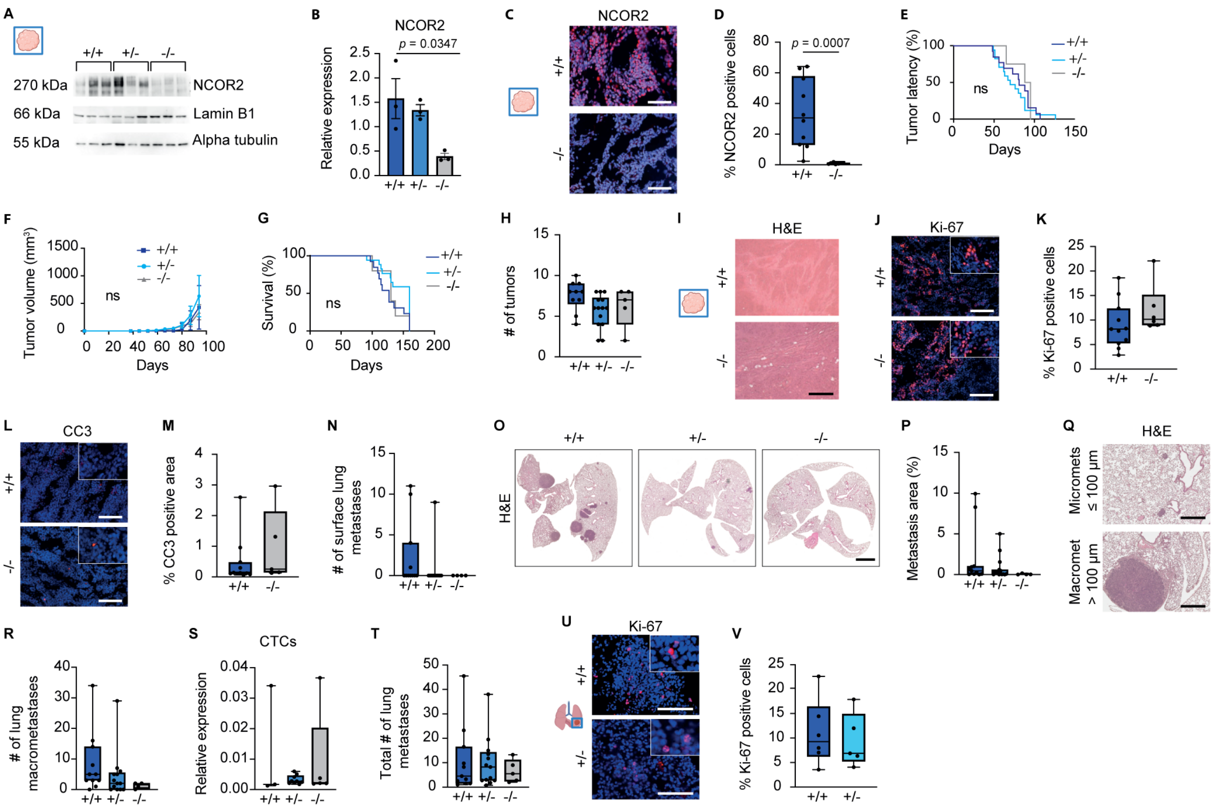

Supplementary Figure 2. NCOR2 knockout diminishes spontaneous lung metastasis of mammary tumor cells in a transgenic mouse model. (A) Representative immunoblots of primary tumors from the MMTV-PyMT transgenic mice (PyMT-NCOR2$^{+/+}$, *n* = 3 mice; PyMT-NCOR2$^{+/-}$, *n* = 3 mice; PyMT-NCOR2$^{-/-}$, *n* = 3 mice) as described in Figure 3A using the indicated antibodies. Lamin B1 and alpha tubulin were used as loading controls (representative data from *n* = 2 independent experiments). (**B**) Bar graphs showing quantification of relative expression of NCOR2 by densitometry for the immunoblots presented in (A) (PyMT-NCOR2$^{+/+}$, *n* = 3 mice; PyMT-NCOR2$^{+/-}$, *n* = 3 mice; PyMT-NCOR2$^{-/-}$, *n* = 3 mice). (**C**) Representative images of immunofluorescence (IF) staining of frozen mammary tumor sections from PyMT-NCOR2$^{+/+}$ and PyMT-NCOR2$^{-/-}$ mice with an antibody specific to NCOR2. Scale bar: 50 µm. (**D**) Box and whisker plots showing quantification of the IF staining shown in (C) (PyMT-NCOR2$^{+/+}$, *n* = 10 mice; PyMT-NCOR2$^{-/-}$, *n* = 5 mice). (**E**) Survival plots displaying tumor latency (days) for MMTV-PyMT mice with wildtype levels (+/+), and heterozygous (+/-) or homozygous (-/-) knockout of NCOR2 in mammary epithelial and tumor cells (PyMT-NCOR2$^{+/+}$, *n* = 13 mice; PyMT-NCOR2$^{+/-}$, *n* = 17 mice; PyMT-NCOR2$^{-/-}$, *n* = 4 mice). (**F**) Mammary tumor outgrowth of the MMTV-PyMT mouse cohorts in (E) (PyMT-NCOR2$^{+/+}$, *n* = 10 mice; PyMT-NCOR2$^{+/-}$, *n* = 14

mice; PyMT-NCOR2$^{-/-}$, n = 4 mice). (**G**) Survival plots related to a predetermined maximal tumor diameter for the MMTV-PyMT mouse cohorts in (E) (PyMT-NCOR2$^{+/+}$, n = 13 mice; PyMT-NCOR2$^{+/-}$, n = 17 mice; PyMT-NCOR2$^{-/-}$, n = 5 mice). (**H**) Box and whisker plots showing quantification of the primary mammary tumor number per mouse for the MMTV-PyMT mouse cohorts in (E) (PyMT-NCOR2$^{+/+}$, n = 10 mice; PyMT-NCOR2$^{+/-}$, n = 14 mice; PyMT-NCOR2$^{-/-}$, n = 5 mice). (**I**) Representative images of hematoxylin and eosin (H&E) stained FFPE mammary tumor sections from PyMT-NCOR2$^{+/+}$ and PyMT-NCOR2$^{-/-}$ mice. (**J**) Representative images of IF staining of frozen mammary tumor sections from PyMT-NCOR2$^{+/+}$ and PyMT-NCOR2$^{-/-}$ mice with an antibody specific to Ki-67. Scale bar: 100 µm. (**K**) Box and whisker plots showing quantification of the IF staining shown in (J) (PyMT-NCOR2$^{+/+}$, n = 11 mice; PyMT-NCOR2$^{-/-}$, n = 6 mice). (**L**) Representative images of IF staining of frozen mammary tumor sections from PyMT-NCOR2$^{+/+}$ and PyMT-NCOR2$^{-/-}$ mice with an antibody specific to cleaved caspase 3 (CC3). Scale bar: 100 µm. (**M**) Box and whisker plots showing quantification of the IF staining shown in (L) (PyMT-NCOR2$^{+/+}$, n = 11 mice; PyMT-NCOR2$^{-/-}$, n = 5 mice). (**N**) Box and whisker plots showing quantification of the number of lung metastases on the surface of the lung for tumor bearing mice (PyMT-NCOR2$^{+/+}$, n = 11 mice; PyMT-NCOR2$^{+/-}$, n = 11 mice; PyMT-NCOR2$^{-/-,}$ n = 4 mice). (**O**) Representative images of hematoxylin and eosin (H&E)-stained FFPE tissue sections derived from the lungs of the MMTV-PyMT mouse cohorts described in (E). Scale bar: 1 mm. (**P**) Box and whisker plots showing quantification of the percentage of metastatic lesion area in relation to total lung area for the mice in (E) (PyMT-NCOR2$^{+/+}$, n = 11 mice; PyMT-NCOR2$^{+/-}$, n = 15 mice; PyMT-NCOR2$^{-/-}$, n = 4 mice). (**Q**) Representative images of H&E stained FFPE lung tissue sections illustrating micrometastases (≤ 100 µm) or macrometastasis (> 100 µm). Scale bar: 200 µm. (**R**) Box and whisker plots showing quantification of the number of lung macrometastases for the mice in (E) (PyMT-NCOR2$^{+/+}$, n = 11 mice; PyMT-NCOR2$^{+/-}$, n = 13 mice; PyMT-NCOR2$^{-/-}$, n = 4 mice). (**S**) Box and whisker plots showing quantification of the relative levels of circulating tumor cells (CTCs) in the MMTV-PyMT mouse cohorts as in (E) (PyMT-NCOR2$^{+/+}$, n = 3 mice; PyMT-NCOR2$^{+/-}$, n = 9 mice; PyMT-NCOR2$^{-/-}$, n = 5 mice). (**T**) Box and whisker plots showing quantification of the total number of lung metastases for the MMTV-PyMT mice in (E) (PyMT-NCOR2$^{+/+}$, n = 11 mice; PyMT-NCOR2$^{+/}$,

n = 13 mice; PyMT-NCOR2$^{-/-}$, *n* = 5 mice). (**U**) Representative images showing immunofluorescence (IF) staining with an antibody specific to Ki-67 in FFPE lung tissue sections from PyMT-NCOR2$^{+/+}$ and PyMT-NCOR2$^{+/-}$ mice. Scale bar: 100 µm. (**V**) Box and whisker plots showing quantification of the IF staining in (U) (PyMT-NCOR2$^{+/+}$, *n* = 6 mice; PyMT-NCOR2$^{+/-}$, *n* = 5 mice). Bar graphs are represented as mean ± s.e.m. using a one-way ANOVA with Tukey's multiple comparisons (B). Box and whisker plots are represented as median (centre line) and interquartile range (box), whiskers indicate minimum to maximum values. Statistical analysis was performed using a two-sided Mann-Whitney U test (D, K, M and V), and Kruskal-Wallis test with Dunn's multiple comparisons (H, N, P and R-T). Line graphs are represented as time series analysis based on two-sided Mann-Whitney U tests (F), or survival curves using a log-rank test (E and G). Abbreviations: ns, not significant. Graphical elements were created with BioRender.com.

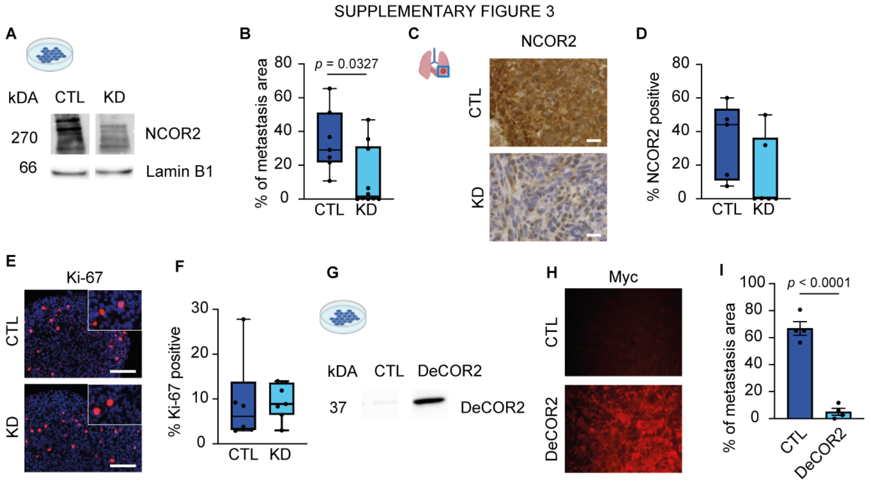

Supplementary Figure 3. Generation of 4T07 breast cancer cells expressing shRNA targeting NCOR2 or the NCOR2-HDAC3 disruptor, DeCOR2 to study experimental lung metastasis. (A) Representative immunoblots using antibodies specific to NCOR2 and Lamin B1 illustrating shRNA-mediated knockdown of NCOR2 expression in 4T07 cells. Lamin B1 was used as a loading control (representative data from n = 3 independent experiments). (**B**) Box and whisker plots quantifying the percentage of metastatic lesion area in relation to total lung area for the mice presented in Figure 4B (CTL, n = 7 mice; KD, n = 10 mice). (**C**) Representative images of immunohistochemical (IHC) staining of FFPE lung tissue sections with an antibody specific to NCOR2. Lung tissues contain metastatic lesions derived from tail vein injections of 4T07 cells expressing a scrambled non-targeting shRNA control (CTL) or an NCOR2-targeting shRNA (KD) as in (A). Scale bar: 20 µm. (**D**) Box and whisker plots showing quantification of positive NCOR2 staining for mouse lung tissues from (C) (CTL, n = 5 mice; KD, n = 6 mice). (**E**) Representative images of immunofluorescence (IF) staining of lung tissue sections containing metastatic lesions, as in (C), with an antibody specific to Ki-67. Scale bar: 100 µm. (**F**) Box and whisker plots showing quantification of the IF staining shown in (E) (CTL, n = 6 mice; KD n = 7 mice). (**G**) Representative immunoblot using an antibody specific to Myc illustrating overexpression of the Myc-tagged DeCOR2 peptide in 4T07 cells in comparison to 4T07 cells expressing an empty vector control (CTL) (representative data from n = 2 independent experiments). (**H**) Representative images of IF staining of 4T07 cells expressing an empty vector (CTL) or Myc-tagged DeCOR2 with an antibody specific to Myc. (**I**) Bar graphs quantifying the percentage of metastatic lesion area in relation to total lung area for the mice presented in Figure 4I (CTL, n = 4 mice; DeCOR2,

$n = 4$ mice). Box and whisker plots are represented as median (centre line) and interquartile range (box), whiskers indicate minimum to maximum values. Statistical analysis was performed using a two-sided Mann-Whitney U test (B, D and F). Bar graphs are represented as mean ± s.e.m using a two-sided unpaired t-test (I). Graphical elements were created with BioRender.com.

SUPPLEMENTARY FIGURE 4

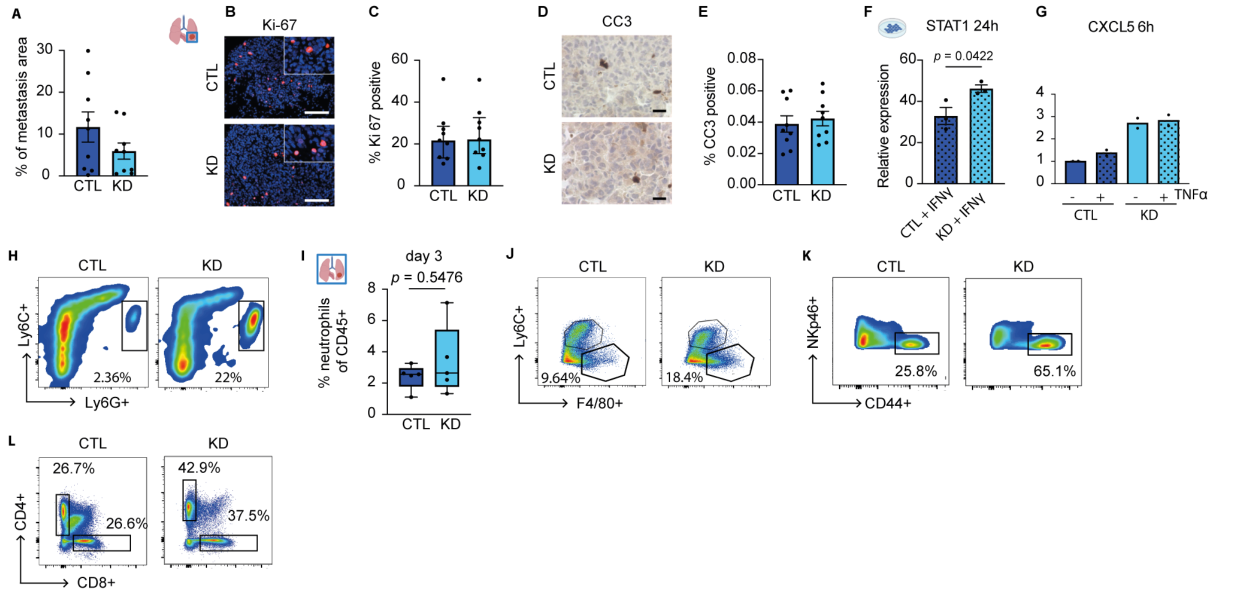

Supplementary Figure 4. NCOR2 regulates inflammation to enable breast cancer cell metastatic colonization of the lung. (**A**) Bar graphs quantifying the percentage of metastatic lesion area in relation to total lung area for the mice presented in Figure 5B (CTL, *n* = 9 mice; KD, *n* = 9 mice). (**B**) Representative images of immunofluorescence (IF) staining of lung tissue sections containing metastatic lesions, as in (A), with an antibody specific to Ki-67. Scale bar: 100 µm. (**C**) Bar graphs showing quantification of the IF staining shown in (B) (CTL, *n* = 9 mice; KD, *n* = 9 mice). (**D**) Representative images of immunohistochemical (IHC) staining of FFPE lung tissue sections, as in (B), with an antibody specific to cleaved caspase 3 (CC3). (**E**) Bar graphs showing quantification of the IHC staining shown in (D) (CTL, *n* = 9 mice; KD, *n* = 9 mice). (**F**) Bar graphs showing quantitative RT-PCR analysis of *STAT1* expression relative to *Gapdh* for 4T07 cells with (KD, *n* = 3 independent experiments) or without (CTL, *n* = 3 independent experiments) knockdown of NCOR2 cultured and stimulated +/- IFNγ for 24 hours. **(G)** Bar graphs showing quantitative RT-PCR analysis of *Cxcl5* expression relative to *Gapdh* for 4T07 cells with (KD, *n* = 2 independent experiments) or without (CTL, *n* = 2 independent experiments) knockdown of NCOR2 cultured and stimulated +/- TNFα for 6 hours. (**H**) Representative flow cytometry plots showing the fraction of CD45-positive cells that also express markers of neutrophils in cells isolated from the lungs of mice following tail vein injection of 4T07 cells with or without knockdown of NCOR2. (**I**) Box and whisker plots showing flow cytometry analysis as in (H) to determine the percentage of CD45-positive cells that are neutrophils at 3 days following tail vein injection of 4T07 cells (CTL, *n* =

5 mice; KD, $n = 5$ mice). (**J**) Representative flow cytometry plots showing the fraction of CD45-positive cells that also express markers of macrophages in cells isolated from the lungs of mice following tail vein injection of 4T07 cells with or without knockdown of NCOR2, corresponding to the experimental design and data presented in Figure 5J and K. **(K)** Representative flow cytometry plots showing the gating strategy to detect CD44-positive NK cells in lung tissues seeded with 4T07 breast cancer cells with (KD) and without (CTL) NCOR2 knockdown, corresponding to the experimental design and data presented in Figure 5M. (**L**) Representative flow cytometry plots showing the gating strategy used to identify CD4+ and CD8+ T cells from lung tissues seeded with 4T07 breast cancer cells with (KD) and without (CTL) NCOR2 knockdown, corresponding to the experimental design and data presented in Figure 5J and N,O. Bar graphs are presented as mean ± s.e.m using a two-sided unpaired t-test (C,E,F). Box and whisker plots are represented as median (centre line) and interquartile range (box), whiskers indicate minimum to maximum values. Statistical analysis was performed using a two-sided Mann-Whitney U test (I). Graphical elements were created with BioRender.com.

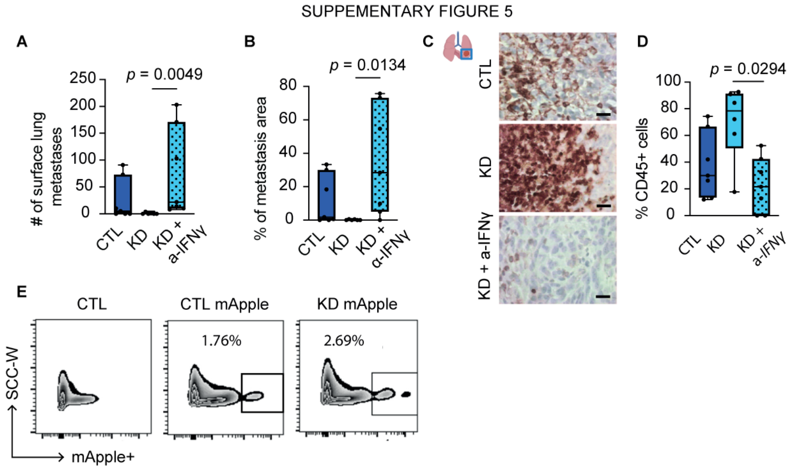

Supplementary Figure 5. NCOR2 knockdown alters mammary tumor cell regulation of T cell responses. (**A**) Analyses correspond to mice presented in Figure 6B showing bioluminescence imaging of lungs 14 days following tail vein injection of 4T07 cells with (KD, *n* = 13 total mice) or without (CTL, *n* = 7 mice) knockdown of NCOR2. Mice receiving cells with NCOR2 KD were subdivided into treatment groups with a control IgG (KD, *n* = 6 mice) or an antibody targeting the IFNγ receptor (KD + a-IFNγ, *n* = 7 mice). Box and whisker plots show quantification of the number of lung metastases detected on the surface of the lungs for mice in Figure 6B. (**B**) Box and whisker plots showing quantification of metastatic lung lesion area relative to total lung area for mice described in (A) and Figure 6B (CTL, *n* = 7 mice; KD, *n* = 6 mice; KD + a-IFNγ, *n* = 7 mice). (**C**) Representative images of immunohistochemical (IHC) staining of FFPE lung tissue sections with an antibody specific to CD45. (**D**) Box and whisker plots showing quantification of the IHC staining shown in (C) (CTL, *n* = 7 mice; KD, *n* = 6 mice; KD + a-IFNγ, *n* = 7 mice). (**E**) Representative flow cytometry plots showing the gating strategy to detect mApple-positive 4T07 breast cancer cells with (KD) and without (CTL) NCOR2 knockdown from mouse lung tissues as described in Figure 6F. Box and whisker plots are represented as median (centre line) and interquartile range (box), whiskers indicate minimum to maximum values. Statistical analysis was performed using a Kruskal-Wallis test with Dunn's multiple comparisons (A, B, and D). Graphical elements were created with BioRender.com.

SUPPLEMENTARY FIGURE 6

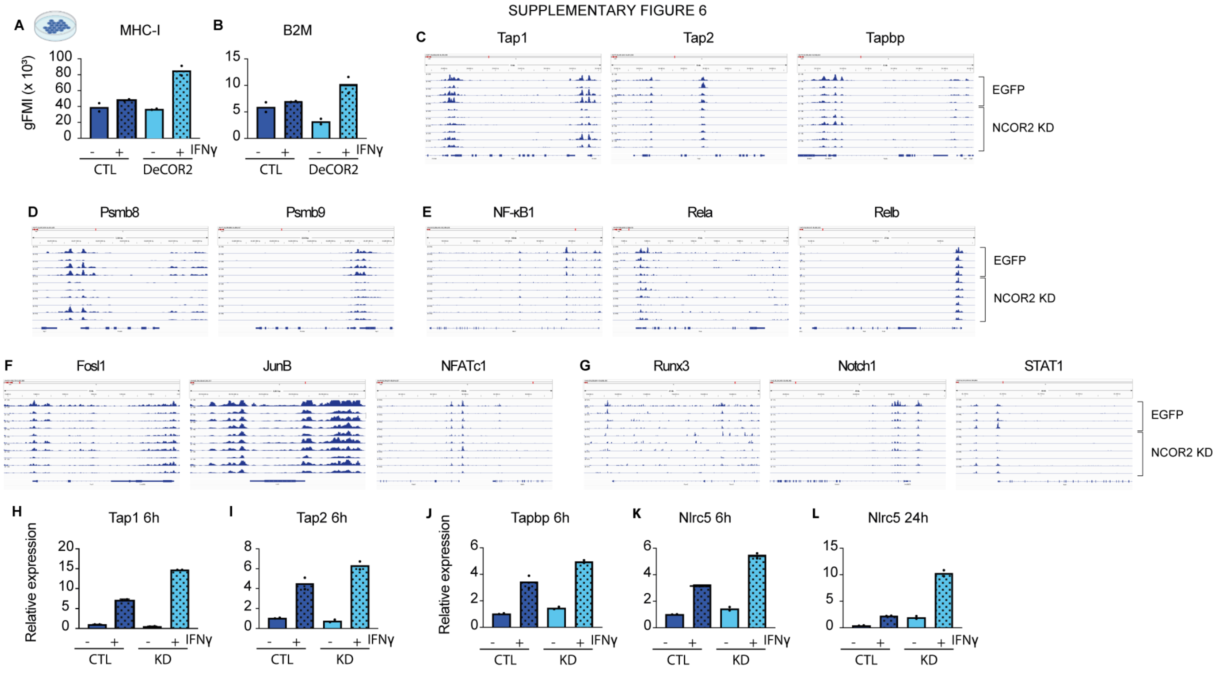

Supplementary Figure 6. Diminished NCOR2 levels or impaired NCOR2-mediated HDAC3 recruitment lead to improved MHC class I molecule presentation on the surface of breast cancer cells. (**A and B**) Flow cytometry analysis of 4T07 cells expressing an empty vector control (CTL, *n* = 2 independent experiments) or the NCOR2-HDAC3 interaction disrupting peptide, DeCOR2 (*n* = 2 independent experiments) cultured and stimulated +/- IFNγ for 48 hours. Bar graphs show quantification of geometric mean fluorescence intensity (gMFI) of MHC-I (A) and β2-microglobulin (B, β2M) detected on the surface of 4T07 cells. (**C-G**) CUT&Tag analysis for illustrating peak profiles comparing genomic occupancy of NCOR2 at the loci of the (C) *Tap1*, *Tap2* and *Tapbp*, (D) *Psmb8* and *Psmb9*, (E) *Nfkb1*, *Rela* and *Relb*, (F) *Fosl1*, *Junb* and *Nfatc1*, (G) *Runx3*, *Notch1* and *Stat1* genes in 4T07 cells with (NCOR2 shRNA, *n* = 6 independent experiments) and without (EGFP shRNA, *n* = 4 independent experiments) NCOR2 knockdown – all compared samples are shown. log2FC = Log2 fold change in expression; p-value obtained using Wilcox Rank Sum test followed by Benjamini Hochberg (BH) correction. (**H-K**) Bar graphs showing quantitative RT-PCR analysis of *Tap1* (H), *Tap2* (I), *Tapbp* (J) and *Nlrc5* (K) expression relative to *Gapdh* for 4T07 cells with (KD, *n* = 2 independent experiments) or without (CTL, *n* = 2 independent experiments) knockdown of NCOR2 cultured and stimulated +/- IFNγ for 6 hours. (**L**) The same analysis as in (H-K) for relative Nlrc5 expression in cultured 4T07 cells +/- IFNγ stimulation for 24 hours. Graphical elements were created with BioRender.com.

Supplementary Table 1. A list of oligonucleotide primers used for qPCR analyses

Primer	Reference ID	Sequence
Tap1 Forward	IDT #484646985	5'- CGT TCT CTA CCA GCT TCA GTT C -3'
Tap1 Reverse	IDT #484646986	5 '- AAG GAG TCC GGT CCA AGT AT -3'
Tap2 Forward	IDT #484646987	5 '- CCA GGA GAA CAG AAC ACT GAT G -3'
Tap2 Reverse	IDT #484646988	5'- GCC ACC ACA AGG AAG AAG AA -3'
Tapbp Forward	IDT #484646983	5'- TGC CAG CCT GAT CTA CAA ATA G -3'
Tapbp Reverse	IDT #484646984	5'- GAC TCT ACG CAT GTG TGT TCT -3'
Nlrc5 Forward	IDT #484646989	5'- CTC ACA GGG CCA AAG ATG ATA G -3'
Nlrc5 Reverse	IDT #484646990	5'- GAG GGA AAT TGT GGA AGG AGA G -3'
Psmb8 Forward	IDT #484646991	5'- GCT ACC CAC AGA GAC AAC TAT TC -3'
Psmb8 Reverse	IDT #484646992	5'- GAC ATC GGA ACT CTC CAC TTT C -3'
Psmb9 Forward	IDT #484646993	5'- GGT TAT GTG GAC GCA GCT TAT -3'
Psmb9 Reverse	IDT #484646994	5'- ACT AGA GCC ATC TCG GTT CA -3'
CXCL2 Forward	IDT #157779098	5'- TCC AGA GCT TGA GTG TGA CG -3'
CXCL2 Reverse	IDT #157779099	5'- CAG GTC AGT TAG CCT TGC CT -3'
Stat1 Forward	IDT #440839952	5'- TCA CAG TGG TTC GAG CTT CAG -3'
Stat1 Reverse	IDT #440839953	5'- GCA AAC GAG ACA TCA TAG GCA- 3'
CCL2 Forward	Primer Bank #141803162c1	5'- TAA AAA CCT GGA TCG GAA CCA AA -3'
CCL2 Reverse	Primer Bank #141803162c1	5'- GCA TTA GCT TCA GAT TTA CGG GT -3'
CXCL10 Forward	Primer Bank #10946576a1	5'- CCA AGT GCT GCC GTC ATT TTC- 3'
CXCL10 Reverse	Primer Bank #10946576a1	5'- GGC TCG CAG GGA TGA TTT CAA -3'
CXCL5 Forward	Primer Bank # 21428236a1	5'- CCG CTG GCA TTT CTG TTG CTG T -3'
CXCL5 Reverse	Primer Bank # 21428236a1	5'- CAG GGA TCA CCT CCA AAT TAG CG -3'
