# Supplementary material for: NCOR2 represses MHC class I molecule expression to drive metastatic progression of breast cancer
Source: Nat Commun. 2026 May 5;17:6067. doi: 10.1038/s41467-026-72168-3 (PMC13351013; doi:10.1038/s41467-026-72168-3)
Supplement: Supplementary file 2 — Description of Additional Supplementary Files [file 41467_2026_72168_MOESM2_ESM.pdf]

Description of Additional Supplementary Files

Supplementary Dataset 1: Combined differential expression analysis of NCOR2 vs EGFP over 0 and 6 hrs of IFNγ treatment. The columns chr, start, end and peak_id contain information for peak identification; The column log2FC summarizes the mean of log2FoldChange_00h and log2FoldChange_06h columns; upstream2kb_genes column contains the gene name that is within 2kB of the corresponding peak and overlap_genes contains the gene names that overlap with the corresponding peak; padj_combined adjusts the p_combined column for multiple hypothesis testing; p_combined is calculated from independent p_value columns using Fisher test.

Supplementary Dataset 2: A list of antibodies used in the study.
