# Supplementary material for: NCOR2 represses MHC class I molecule expression to drive metastatic progression of breast cancer
Source: Nat Commun. 2026 May 5;17:6067. doi: 10.1038/s41467-026-72168-3 (PMC13351013; doi:10.1038/s41467-026-72168-3)
Supplement: Supplementary file 5 — Reporting Summary [file 41467_2026_72168_MOESM5_ESM.pdf]

nature portfolio

Corresponding author(s): Valerie Weaver, PhD

Last updated by author(s): Feb 20, 2026

Reporting Summary

Nature Portfolio wishes to improve the reproducibility of the work that we publish. This form provides structure for consistency and transparency in reporting. For further information on Nature Portfolio policies, see our Editorial Policies and the Editorial Policy Checklist.

Statistics

For all statistical analyses, confirm that the following items are present in the figure legend, table legend, main text, or Methods section.

n/a	Confirmed	
☐	☒	The exact sample size (*n*) for each experimental group/condition, given as a discrete number and unit of measurement
☐	☒	A statement on whether measurements were taken from distinct samples or whether the same sample was measured repeatedly
☐	☒	The statistical test(s) used AND whether they are one- or two-sided<br>*Only common tests should be described solely by name; describe more complex techniques in the Methods section.*
☒	☐	A description of all covariates tested
☐	☒	A description of any assumptions or corrections, such as tests of normality and adjustment for multiple comparisons
☐	☒	A full description of the statistical parameters including central tendency (e.g. means) or other basic estimates (e.g. regression coefficient) AND variation (e.g. standard deviation) or associated estimates of uncertainty (e.g. confidence intervals)
☐	☒	For null hypothesis testing, the test statistic (e.g. *F*, *t*, *r*) with confidence intervals, effect sizes, degrees of freedom and *P* value noted<br>*Give P values as exact values whenever suitable.*
☒	☐	For Bayesian analysis, information on the choice of priors and Markov chain Monte Carlo settings
☐	☒	For hierarchical and complex designs, identification of the appropriate level for tests and full reporting of outcomes
☐	☒	Estimates of effect sizes (e.g. Cohen's *d*, Pearson's *r*), indicating how they were calculated

Our web collection on statistics for biologists contains articles on many of the points above.

Software and code

Policy information about availability of computer code

Data collection	Flow data was acquired using BD FACSDiva software version 9.0<br>Bioluminescence images were acquired using Live Imaging Software v.4.5.2 (PerkinElmer)<br>Histology slides were scanned using Aperio ImageScope software v12.2.2.5015<br>Images of Immunohistochemistry were captured using Infinity Capture v6.5.6 software<br>Immunofluorescence images were captured using NIS-Elements software v5.41.02<br>Quantitative RT-PCR (qRT-PCR) results were recorded with a LightCycler System (Roche) according to the manufacturer's instructions.<br>Publicly available signle-cell datasets were downloaded from the Gene Expression Omnibus (GEO) database entry GSE161529.
Data analysis	For analysis of single cell RNAseq data, the Seurat pipeline was applied to each sample. Raw counts of annotated cancer cells were normalized (Seurat NormalizeData function) and scaled (ScaleData function. The expression of selected genes then visualized in heatmaps using the R package ComplexHeatmap. R script used for the analysis of single cell datasets is available at https://github.com/eshenderov/Breast-Single-Cell-Analysis.<br>For analysis of CUT&Taq sequencing data, sequences from dual index demultiplexed libraries were processed using tools available on www.usegalaxy.org. Nextera Adapter sequences were removed from Read 1 and 2 using Cutadapt. Bowtie2 was used to map paired end reads to the Mouse (mus musculus):mm10 build using parameters: –fr for valid mate pairs, Very sensitive end-to-end (--very-sensitive) for Analysis mode and allowing Read1 and Read2 dovetailing. The resulting BAM file was filtered to include only proper pairs with Mapquality phred scores =>30 and excluding those mapping to the mitochondrial genome. Duplicates were removed using MarkDuplicates scoring on the basis of SUM_OF_BASE_QUALITIES, and Optical Duplicates set at 100 pixels. BAM files were converted to Bigwig and bigwigAverage was used to find the normalized average of technical repeats of the NCOR2 knockdown vs EGFP shRNA control.<br>All the code used for the analysis of CUT&Tag samples is available at https://github.com/eshenderov/CUTNTag.

Bioluminescence imaging was analyzed using Live Imaging Software v.4.5.2 (PerkinElmer)
Immunohistochemistry was analyzed using QuPath software v0.4.3
Immunofluorescence images were analyzed using FIJI v2.14.0/1.54f
Flow data was analyzed using FlowJo™ software v10.8
Statistical analysis was conducted using GraphPad Prism Version 10.0.2

For manuscripts utilizing custom algorithms or software that are central to the research but not yet described in published literature, software must be made available to editors and reviewers. We strongly encourage code deposition in a community repository (e.g. GitHub). See the Nature Portfolio guidelines for submitting code & software for further information.

Data

Policy information about availability of data

All manuscripts must include a data availability statement. This statement should provide the following information, where applicable:

- Accession codes, unique identifiers, or web links for publicly available datasets
- A description of any restrictions on data availability
- For clinical datasets or third party data, please ensure that the statement adheres to our policy

CUT&Tag data generated in is study is made available at GSE320158 . All the code used for the analysis of CUT&Tag samples is available at https://github.com/eshenderov/CUTNTag. Publicly available single-cell RNA sequencing datasets were downloaded from GSE161529. R script used for the amalysis of single cell datasets is available at https://github.com/eshenderov/Breast-Single-Cell-Analysis.

Research involving human participants, their data, or biological material

Policy information about studies with human participants or human data. See also policy information about sex, gender (identity/presentation), and sexual orientation and race, ethnicity and racism.

Reporting on sex and gender	Human breast cancer specimens and patient derived xenografts used for studies were all from individuals of the female sex. Gender was not specified for human specimens, so no information on gender is reported.
Reporting on race, ethnicity, or other socially relevant groupings	No bias in selection based on race or ethnicity or other socially relevant groupings were used to make decisions on inclusion.
Population characteristics	No selection criteria based on age was used for human research subjects. All subjects were female and many had matched lymph node metastases as indicated. Other patient characteristics for Patient derived xenograft tissues and gene expression and single cell RNAseq analyses have been previously reported. Patients that had received neoadjuvant chemoterapy versus those that had not were also used in the analysis.
Recruitment	Some patients were recruited based on parameters of having received neoadjuvant chemotherapy or not for comparison. a commercially available dataset was prepared to collect specimens of primary breast cancer with matched lymph node metastasis (US Biomax, Rockville, MD, USA). Other datasets for single cell RNA seq and gene expression analyses were previously published and publicly available.
Ethics oversight	All human breast tissue specimens were collected prospectively from consenting patients (informed consent provided prior to surgery by the involved surgeons and pathologiest) undergoing surgery at the University of California, San Francisco, (UCSF) or Duke University Medical Center between 2010 and 2020. Samples were collected, stored and analyzed with deidentified labels to protect patient data in accordance with the procedures outlined in the Institutional Review Board Protocols #10-03832 and #10-05046 approved by the UCSF Committee of Human Resources, and the Duke University IRB (Pro00054515).

Note that full information on the approval of the study protocol must also be provided in the manuscript.

Field-specific reporting

Please select the one below that is the best fit for your research. If you are not sure, read the appropriate sections before making your selection.

☒ Life sciences ☐ Behavioural & social sciences ☐ Ecological, evolutionary & environmental sciences

For a reference copy of the document with all sections, see nature.com/documents/nr-reporting-summary-flat.pdf

Life sciences study design

All studies must disclose on these points even when the disclosure is negative.

Sample size	Sample sizes in mouse studies were determined based on previously published data demonstrating statistically significant differences in tumor progression and immune response using orthotopic tumor xenografts, syngeneic mouse models established with transformed mammary cells genetically engineered to express short hairpin RNA and DeCOR2, and transgenic mouse models expressing MMTV-PyMT. Sample sizes for evaluating clinical studies were determined based on statistically significant differences in factors impacting tumor aggressiveness, i.e., neoadjuvant chemotherapy, as well as on sample availability. Identification and statistical analysis of the differentially expressed peaks in the CUT & Tag experiment were performed using DESeq2 package in R 4.4.0. The combined p-value after multiple

hypothesis correction was obtained from a total of 6 NCOR2 knockdown samples and 4 EGFP scambled vectors as a control. A total of 5 background control samples (IgG control) were used to model the background during peak calling with MACS3.
Tsai, K.K. et al. Screening of organoids derived from patients with breast cancer implicates the repressor NCOR2 in cytotoxic stress response and antitumor immunity. Nat Cancer 3, 734–752 (2022).

Chan, T.S. et al. Metronomic chemotherapy prevents therapy-induced stromal activation and induction of tumor-initiating cells. J Exp Med 213, 2967–2988 (2016).

Gross, E.T. et al. Immunosurveillance and immunoediting in MMTV-PyMT-induced mammary oncogenesis. Oncoimmunology 6, e1268310 (2017).

Zawati, I. et al. Can residual proliferative cancer burden predict long-term outcomes following neoadjuvant chemotherapy in breast cancer? Pathology (2025).

Data exclusions: All data was included with the exception of animals that succumbed to tumor burden and died prior to completion of the experiment. These animals were excluded.

Replication: For all the findings identified in our study, the experiments were replicated a minimum of two times with similar results.

Randomization: For all analyses, samples were randomized. and mouse littermates were evenly distributed across different experimental groups to avoid any potential bias.

Blinding: Patient and mouse samples were monitored by unique identifiers to ensure investigators were blinded for all studies.

Reporting for specific materials, systems and methods

We require information from authors about some types of materials, experimental systems and methods used in many studies. Here, indicate whether each material, system or method listed is relevant to your study. If you are not sure if a list item applies to your research, read the appropriate section before selecting a response.

Materials & experimental systems

n/a	Involved in the study	
☐	☒	Antibodies
☐	☒	Eukaryotic cell lines
☒	☐	Palaeontology and archaeology
☐	☒	Animals and other organisms
☒	☐	Clinical data
☒	☐	Dual use research of concern
☒	☐	Plants

Methods

n/a	Involved in the study	
☒	☐	ChIP-seq
☐	☒	Flow cytometry
☒	☐	MRI-based neuroimaging

Antibodies

Antibodies used: The antibodies are commercially available and validated by the manufacturers. This information is included in Supplementary Table 2.

Primary antibodies for immunohistochemistry and immunofluorescence

- CD8a Invitrogen #MA5-14548 1:100 SP16
- CD45 BD Pharmingen #AB_394606 1:200 30-F11
- cleavedcaspase-3 Cell Signaling #9661 1:200 polyclonal (Asp175)
- Ki-67 Sigma Aldrich #SAB5700770 1:200 polyclonal
- NCOR2 Sigma Aldrich #SAB4503680 1:200 polyclonal

Flow cytometry panel for neutrophils

- CD45 Pacific Blue Biolegend #103125 1:400 30-F11
- CD11b PECy7 eBioscience #25-0112-82 1:200 M1/70
- CD45R APCeFluor780 eBioscience #47-0452-82 1:200 RA3-6B2
- Ly6C BV711 Biolegend #128037 1:400 HK1.4
- Ly6G APC eBioscience #17-5931-81 1:400 RB6-8C5

Flow cytometry panel for macrophages, monocytes, CD4 T cells, and CD8 T cells

- CD25 FITC Biolegend #102008 1:200 PC61
- Ly6G PECy7 Invitrogen #25-9668-82 1:400 1A8-Ly6g
- CD39 PEDazzle Biolegend #143812 1:400 Duha59
- CD11b APC eBioscience #17-0112-82 1:100 M1/70
- CD90.2 AF700 Biolegend #105328 1:400 30-H12
- CD8 PerCp-Cy5.5 Biolegend #100734 1:400 53-6.7

- CD45 BV421 eBioscience #48-0451-82 1:400 30-F11
- F4/80 BV510 BD Biosciences #569615 1:400 T45-2342
- BV605 BD Biosciences #563059 1:400 J43
- CD69 BV650 BD Biosciences #569688 1:400 H1.2F3
- LyC BV711 BD Biosciences #755195 1:400 HK1.4
- CD45R BV785 Biolegend #103246 1:400 RA3-6B2
- CD4 BUV395 BD Biosciences #565974 1:400 GK1.5
- CD44 BUV737 Biolegend #103037 1:400 IM7

Flow cytometry panel for activated NK cells

- CD45 Pacific Blue Biolegend #103127 1:400 30-F11
- NKp46 PE eBioscience # 12-3351-80 1:200 29A1.4
- CD44 BV650 Biolegend #103049 1:400 IM7

Flow cytometry for MHC class I and b2-microglobulin cell surface expression

- TruStainFcX™ (antimouse CD16/32) Fc Block Biolegend #101320 N/A (1.0 μg per 10^6 cells in 100 μl volume)
- PE antimouse H-2Kd/H-2Dd (MHC Class I) Biolegend #114708 1:400 34-1-2S
- PE/Cyanine7 antimouse β2-microglobulin Biolegend #154508 1:400 A16041A

Immunoblots and CUT&Tag

- NCOR2 Sigma Aldrich #06-891 1:1000 polyclonal https://www.sigmaaldrich.com/CZ/cs/product/mm/06891
- NCOR2 Sigma Aldrich #06-891 1:200 polyclonal https://www.sigmaaldrich.com/CZ/cs/product/mm/06891

In vivo treatments

- Rat IgG1 Isotype Control Leinco Technologies #R1379 N/A (500 μg i.p. per mouse) GL113
- antimouse IFNγ Leinco Technologies Cat# I-1209 N/A (500 μg i.p. per mouse) XMG1.2

Secondary antibodies

- Goat anti-Rabbit IgG (H+L), Alexa Fluor 488ThermoFisherScientific #A-11008 1:500 Polyclonal
- Guinea pig anti-Rabbit IgG (H+L) Novus Biologicals # NBP1-72763 1:200 Polyclonal

Validation

All antibodies have been validated for use in their respective applications (western blot, immunohistochemistry, immunofluorescence, and flow cytometry), as reported on the manufacturers' product pages. Moreover, for immunohistochemistry and immunofluorescence and flow cytometry, secondary only and isotype controls were used as required to validate antibody specificity.This information is included in Sullpementary Table 2.

Primary antibodies for immunohistochemistry and immunofluorescence

- CD8a Invitrogen #MA5-14548 1:100 SP16 https://www.thermofisher.com/antibody/product/CD8-Antibody-clone-SP16-Monoclonal/MA5-14548

- CD45 BD Pharmingen #AB_394606 1:200 30-F11 https://www.bdbiosciences.com/enat/products/reagents/functional-cell-basedreagents/purified-rat-anti-mousecd45.553076?tab=product_details

- cleavedcaspase-3 Cell Signaling #9661 1:200 polyclonal (Asp175) https://www.cellsignal.com/products/primaryantibodies/cleaved-caspase-3-asp175-antibody/9661?srsltid=AfmBOopnHuh1ILCNGb7EptB0hrfdaJuq7oPeogzaOuP74wIfUsEYO9IH

- Ki-67 Sigma Aldrich #SAB5700770 1:200 polyclonal https://www.sigmaaldrich.com/CZ/cs/productsigmasab5700770srsltid=AfmBOorlgH2mS3ErFpJQ_o1ThbbjZPYLMtzlC3L5buasQIWRIdWSZK7N

- NCOR2 Sigma Aldrich #SAB4503680 1:200 polyclonal https://www.sigmaaldrich.com/CZ/cs/product/sigma/sab4503680

Flow cytometry panel for neutrophils

- CD45 Pacific Blue Biolegend #103125 1:400 30-F11 https://www.biolegend.com/enie/products/pacific-blue-anti-mouse-cd45-antibody-3102

- CD11b PECy7 eBioscience #25-0112-82 1:200 M1/70 https://www.thermofisher.com/antibody/product/CD11b-Antibody-clone-M1-70-Monoclonal/25-0112-82

- CD45R APCeFluor780 eBioscience #47-0452-82 1:200 RA3-6B2 https://www.thermofisher.com/antibody/product/CD45R-B220-Antibody-clone-RA3-6B2-Monoclonal/47-0452-82

- Ly6C BV711 Biolegend #128037 1:400 HK1.4 https://www.biolegend.com/enie/products/brilliant-violet-711-anti-mousely-6c-antibody-8935

- Ly6G APC eBioscience #17-5931-81 1:400 RB6-8C5 https://www.thermofisher.com/order/genomedatabase/dataSheetPdf?producttype=antibody&productsubtype=antibody_primary&productId=17-5931-81&version=156

Flow cytometry panel for macrophages, monocytes, CD4 T cells, and CD8 T cells

- CD25 FITC Biolegend #102008 1:200 PC61 https://www.biolegend.com/enie/products/pe-anti-mouse-cd25-antibody-424

- Ly6G PECy7 Invitrogen #25-9668-82 1:400 1A8-Ly6g https://www.biocompare.com/9776-Antibodies/14501118-Ly-6G-Monoclonal-Antibody-1A8-Ly6g-PE-Cyanine7-eBioscience-8482/

- CD39 PEDazzle Biolegend #143812 1:400 Duha59 https://www.biolegend.com/frch/products/pe-dazzle-594-anti-mousecd39-antibody-16385?displayInline=true&filename=PE/Dazzle™%20594%20antimouse%20CD39%20Antibody.pdf&leftRightMargin=15&pdf=true&topBottomMargin=15&v=20241102123737

- CD11b APC eBioscience #17-0112-82 1:100 M1/70 https://www.thermofisher.com/antibody/product/CD11b-Antibody-clone-M1-70-Monoclonal/17-0112-82

- CD90.2 AF700 Biolegend #105328 1:400 30-H12 https://www.biolegend.com/enie/products/apc-cyanine7-anti-mousecd90-2-thy1-2-antibody-6671

- CD8 PerCp-Cy5.5 Biolegend #100734 1:400 53-6.7 https://www.biolegend.com/nlnl/products/percp-cyanine5-5-anti-mousecd8a-antibody-4255?displayInline=true&filename=PerCPCyanine5.5%20antimouse%20CD8a%20Antibody.pdf&leftRightMargin=15&pdf=true&topBottomMargin=15&v=20250227010954

- CD45 BV421 eBioscience #48-0451-82 1:400 30-F11 https://www.thermofisher.com/antibody/product/CD45-Antibody-clone-30-F11-Monoclonal/48-0451-82

- F4/80 BV510 BD Biosciences #569615 1:400 T45-2342 https://www.fishersci.com/shop/products/f4-80-rat-anti-mouse-bv510-clone-t45-2342-bdhorizon/BDB569615#?keyword=#569615PD1

- BV605 BD Biosciences #563059 1:400 J43 https://www.fishersci.com/shop/products/anti-cd279-bv605-clone-j43-bd/BDB563059 searchHijack=true&searchTerm=%23563059&searchType=RAPID&matchedCatNo=%23563059

- CD69 BV650 BD Biosciences #569688 1:400 H1.2F3 https://www.fishersci.com/shop/products/bv650-hamster-anti-mouse-cd69-bdhorizon/BDB569688

- LyC BV711 BD Biosciences #755195 1:400 HK1.4 https://www.fishersci.com/shop/products/ly-6c-rat-anti-mouse-bv711-clone-hk1-4-rmab-also-known-as-hk1-4-bdoptibuild/BDB755195?searchHijack=true&searchTerm=%23755195&searchType=RAPID&matchedCatNo=%23755195

- CD45R BV785 Biolegend #103246 1:400 RA3-6B2 https://www.biolegend.com/enie/products/brilliant-violet-785-anti-mousehuman--cd45r-b220-antibody-7960

- CD4 BUV395 BD Biosciences #565974 1:400 GK1.5 https://www.fishersci.com/shop/products/cd4-rat-anti-mouse-buv395-clone-gk1-5-bd-horizon/BDB565974

- CD44 BUV737 Biolegend #103037 1:400 IM7 https://www.biolegend.com/enie/products/brilliant-violet-570-anti-mousehuman-cd44-antibody-7386

Flow cytometry panel for activated NK cells

- CD45 Pacific Blue Biolegend #103127 1:400 30-F11 https://www.biolegend.com/enie/products/alexa-flβor-700-anti-mousecd45-antibody-3407

-- NKp46 PE eBioscience # 12-3351-80 1:200 29A1.4 https://www.thermofisher.com/antibody/product/CD335-NKp46-Antibody-clone-29A1-4-Monoclonal/12-3351-80

- CD44 BV650 Biolegend #103049 1:400 IM7 https://www.biolegend.com/enus/products/brilliant-violet-650-antimouse-human-cd44-antibody-8923

Flow cytometry for MHC class I and b2-microglobulin cell surface expression

- TruStainFcX™ (antimouse CD16/32) Fc Block Biolegend #101320 N/A (1.0 µg per 10^6 cells in 100 µl volume) https://www.biolegend.com/fr-ch/products/trustain-fcxanti-mouse-cd16-32-antibody-5683?displayInline=true&filename=TruStain%20FcX™%20(antimouse%20CD1632)%20Antibody.pdf&leftRightMargin=15&pdf=true&topBottomMargin=15&v=20250227010954

- PE antimouse H-2Kd/H-2Dd (MHC Class I) Biolegend #114708 1:400 34-1-2S https://d1spbj2x7qk4bg.cloudfront.net/frch/products/pe-anti-mouse-h-2kd-h-2dd-antibody-1887?pdf=true&displayInline=true&leftRightMargin=15&topBottomMargin=15&filename=PE 20antimouse%20H-2K%3CSUP%3Ed%3C/SUP%3E/H-2D%3CSUP%3Ed%3C/SUP%3E%20Antibody.pdf&v=20230425063019

- PE/Cyanine7 antimouse β2-microglobulin Biolegend #154508 1:400 A16041A https://d1spbj2x7qk4bg.cloudfront.net/frch/products/pe-cyanine7-anti-mouse-beta2-microglobulin-antibody-15411?pdf=true&displayInline=true&leftRightMargin=15&topBottomMargin=15&filename=PE/Cyanine7%20aulin nti-mouse%20%CE%B22-microglobulin%20Antibody.pdf&v=20230726063409

Immunoblots and CUT&Tag

- NCOR2 Sigma Aldrich #06-891 1:1000 polyclonal https://www.sigmaaldrich.com/CZ/cs/product/mm/06891

- NCOR2 Sigma Aldrich #06-891 1:200 polyclonal https://www.sigmaaldrich.com/CZ/cs/product/mm/06891

In vivo treatments

- Rat IgG1 Isotype Control Leinco Technologies #R1379 N/A (500 µg i.p. per mouse) GL113 https://www.leinco.com/p/rat-igg1-gl113-isotypecontrol-purified-functional-grade-platinum/

- antimouse IFNγ Leinco Technologies Cat# I-1209 N/A (500 µg i.p. per mouse) XMG1.2 https://www.leinco.com/p/anti-mouse-ifnγ clone-xmg1-2-purified-in-vivo-platinum-functional-grade/

Secondary antibodies

- Goat anti-Rabbit IgG (H+L), Alexa Fluor 488ThermoFisherScientific #A-11008 1:500 Polyclonal https://www.thermofisher.com/antibody/product/Goatanti-Rabbit-IgG-H-L-Cross-Adsorbed-Secondary-Antibody-Polyclonal/A-11008

- Guinea pig anti-Rabbit IgG (H+L) Novus Biologicals # NBP1-72763 1:200 Polyclonal https://www.novusbio.com/products/igg-h-lantibody_nbp1-72763?srsltid=AfmBOor5AAdT1PBhIFu0YIa6h9J0dNcM3PoEurvfUuTWM_4zOqwAM7dC

Eukaryotic cell lines

Policy information about cell lines and Sex and Gender in Research

Cell line source(s)	4T1 and 4TO7 murine breast cancer cells were obtained from the ATCC. These cell lines are from female mice. Human triple-negative Patient Derived Xenograft (PDX) line BCM2665 was obtained from Dr. Alana Welm at the Huntsman Cancer Institute, University of Utah, Utah.
Authentication	Cell lines were authenticated by STR profiling
Mycoplasma contamination	All cell lines tested negative for mycoplasma
Commonly misidentified lines (See ICLAC register)	None

Animals and other research organisms

Policy information about studies involving animals; ARRIVE guidelines recommended for reporting animal research, and Sex and Gender in Research

Laboratory animals	NCOR2 (SMRT) conditional knockout mice (floxed) were provided by Dr. Mitchel Lazar and backcrossed onto an FVBN/J background (Strain# 001800)<br>MMTV-cre (Tg(MMTV-cre)4Mam/J; strain# 003553) and MMTV-PyMT (Tg(MMTV-PyVT)634Mul/J; strain 002374) mice were obtained from colleagues at UCSF and maintained on an FVBN/J background and crossed with SMRT-floxed mice. Tumor development was monitored weekly and mice were sacrificed when tumors reached 1.5cm in diameter.<br>BALBC/J (Strain# 000651) mice were purchased from The Jackson laboratory and tail vein and orthotopic injections were performed in mice at 6-8 and 16 weeks of age respectively.<br>NSG mice were purchased from The Jackson Laboratory and tail vein injections were performed in mice at 29 weeks of age.<br>Mice were maintained in pathogen-free, ventilated HEPA-filtered cages under stable housing conditions of 30-70% humidity, a temperature of 20-26 degrees Celsius, and a 12:12 hour dark:light cycle.
Wild animals	No wild animals were used.
Reporting on sex	All mice used for the study of breast cancer primary tumor growth and spontaneous or experimental metastatic progression in the lung were female mice.
Field-collected samples	No field collected samples were used.
Ethics oversight	Animal husbandry and all procedures on mice were carried out in Laboratory Animal Resource Center (LARC) facilities at UCSF Parnassus in accordance with the guidelines stipulated by the Institutional Animal Care Use Committee (IACUC) protocol, #AN207099, which adheres to the NIH Guide for the Care and Use of Laboratory Animals.

Note that full information on the approval of the study protocol must also be provided in the manuscript.

Plants

Seed stocks

Report on the source of all seed stocks or other plant material used. If applicable, state the seed stock centre and catalogue number. If plant specimens were collected from the field, describe the collection location, date and sampling procedures.

Novel plant genotypes

Describe the methods by which all novel plant genotypes were produced. This includes those generated by transgenic approaches, gene editing, chemical/radiation-based mutagenesis and hybridization. For transgenic lines, describe the transformation method, the number of independent lines analyzed and the generation upon which experiments were performed. For gene-edited lines, describe the editor used, the endogenous sequence targeted for editing, the targeting guide RNA sequence (if applicable) and how the editor was applied.

Authentication

Describe any authentication procedures for each seed stock used or novel genotype generated. Describe any experiments used to assess the effect of a mutation and, where applicable, how potential secondary effects (e.g. second site T-DNA insertions, mosiacism, off-target gene editing) were examined.

Flow Cytometry

Plots

Confirm that:

☒ The axis labels state the marker and fluorochrome used (e.g. CD4-FITC).

☒ The axis scales are clearly visible. Include numbers along axes only for bottom left plot of group (a 'group' is an analysis of identical markers).

☒ All plots are contour plots with outliers or pseudocolor plots.

☒ A numerical value for number of cells or percentage (with statistics) is provided.

Methodology

Sample preparation

Mouse lung tissue was thawed and chopped with a razor blade. Chopped tissue was digested in 100 U ml−1 collagenase type 1 (Worthington Biochemical, catalogue #: LS004196), 500 U ml−1 collagenase type 4 (Worthington Biochemical, catalog #: LS004188) and 200 μg ml−1 DNase I (Roche, catalog #: 10104159001) in DMEM while shaking at 37 °C. Digested tissue was filtered using a 100μm filter to remove remaining pieces. Red blood cells were lysed in ACK buffer (Thermo Fisher Scientific, catalog #: A1049201) and remaining cells were counted. Samples were then washed with FACS buffer (2% FBS in PBS) and resuspended in appropriate buffer for staining for flow cytometric analysis.
For flow cytometric analyses, cells were washed with PBS prior to staining with Zombie NIR Fixable live/dead dye (Biolegend, Catalog #: 423117) for 20 min at 4°C. Cells were washed in PBS followed by surface staining for 30 min at 4°C with directly conjugated antibodies diluted in FACS buffer containing anti-CD16/32 (clone 2.4G2; BioXCell, catalog #: BP0307) to block non-specific binding
For flow cytometry analysis of cell death, MHC class I and Beta2-microglobulin cell surface expression in cells in culture, 4T07 cells expressing IPTG-inducible NCOR2-targeting shRNAs or GFP-targeting shRNA were cultured as described and treated with IPTG for 72 hrs prior to the reapplication of IPTG (1 mM) and stimulation with and without IFNgamma for 48 hrs. For quantification of cell death, cell media was collected and attached cells were harvested by trypsinization for staining with the Live-or-Dye NucFix™ Red Staining Kit (Biotium, catalog: #: 32010-T) for 30 mins prior to two washes with PBS and fixation in 2% paraformaldehyde (PFA). For assays examining cell surface expression of MHC class I molecules and beta2-microglobulin, cells were treated in the same manner as above but also co-treated with the caspase inhibitors Ac-DEVD-CHO and Ac-IETD-CHO for 24 hrs prior to and throughout the duration of the experiment to avoid loss of cells due to apoptosis. Following treatment, cell media was collected and attached cells were harvested by trypsinization for blocking in PBS+2% Fetal bovine serum (FBS; FACS wash buffer), mouse serum and TruStain FcX™ (anti-mouse CD16/32) Antibody (BioLegend; catalog #: 101320)

Instrument

BD LSRFortessa™ Cell Analyzer (BD Biosciences)

Software

BD FACSDiva™ software v9.0 for acquisition and FlowJo™ v10.8 Software for cell population and expression analysis

Cell population abundance

To determine immune cell population abundance, the following antibodies were used: anti-CD25 FITC (Biolegend, catalog #: 101907, dilution 1:200, clone 3C7), anti-CD11b APC (eBioscience, catalog #: 17-0112-82, dilution 1:100, clone M1/70), anti-CD90.2 AF700 (Biolegend, catalog #: 105319, dilution 1:400, clone 30-H12), anti-CD8 PerCp-Cy7 (Biolegend, catalog #: 100733, dilution 1:400, clone 53-6.7), anti-CD45 BV421 (eBioscience, catalog #: 404-0451-82, dilution 1:400, clone 30-F11), anti-CD45R BV785 (Biolegend, catalog #: 103245, dilution 1:400, clone RA3-6B2), anti-CD4 BUV395 (Biolegend, catalog #: 344627, dilution 1:400, clone RM4-5), anti-CD44 BUV737 (Biolegend, catalog #: 103077, dilution 1:400, clone IM7), Ly6G PECy7 (Invitrogen, #25-9668-82 1:400), CD39 PEDazzle (BioLegend, #143804 1:400), Live/dead APC-Cy7 (Invitrogen, #L34992 1:1000), F4/80 BV510 (BD Biosciences, #569615 1:400), CD69 BV650 (BD Biosciences, #569688 1:400), Ly6C BV711 (BD Biosciences, #755195 1:400), CD45 Pacific Blue (Biolegend, #103127 1:400), NKp46 PE (eBioscience, #312-3351-80 1:200), CD44 BV650 (Biolegend, #103033 1:400), Live/dead Fixable Blue (Invitrogen, #L34961 1:1000), CD11b PECy7 (eBioscience, #25-0112-82 1:200), CD45R APCeFluor780 (eBioscience, #47-0452-82 1:200), Ly6C BV711 (Biolegend, #128037 1:400), Ly6G APC (eBioscience, #17-5931-81 1:400), PD1 BV605 (BD Biosciences, #563059 1:400). Cell population abundance was determined using the gating strategy described in the Source data as well as histograms of expression and geometric mean fluorescence intensity to compare CD44 abundance in different cell populations.

For cell death and surface level expression of MHC class I and beta2-microglobulin, the following antibodies were used: an

anti-mouse H-2Kd/H-2Dd PE Antibody (MHC Class I, clone 34-1-2S, BioLegend; catalog #: 114708, 1:200) and an anti-mouse β2-microglobulin PE/Cyanine7 Antibody (clone A16041A, catalog #: 154508, 1:200).
Surface level expression was determined using a histogram of expression and geometric mean fluorescence intensity to compare cell culture conditions.

Gating strategy	Gating strategies used for flow cytometry is presented in the source data files.

☒ Tick this box to confirm that a figure exemplifying the gating strategy is provided in the Supplementary Information.
