# Supplementary material for: NCOR2 represses MHC class I molecule expression to drive metastatic progression of breast cancer
Source: Nat Commun. 2026 May 5;17:6067. doi: 10.1038/s41467-026-72168-3 (PMC13351013; doi:10.1038/s41467-026-72168-3)
Supplement: Supplementary file 6 — Transparent Peer Review file [file 41467_2026_72168_MOESM6_ESM.pdf]

nature portfolio

Peer Review File

NCOR2 represses MHC class I molecule expression to drive metastatic progression of breast cancer

Corresponding Author: Dr Valerie Weaver

This manuscript has been previously reviewed at another journal. This document only contains information relating to versions considered at Nature Communications.

This file contains all reviewer reports in order by version, followed by all author rebuttals in order by version.

Version 0:

Reviewer comments:

Reviewer #1

(Remarks to the Author)
The authors have addressed previous comments.

Reviewer #3

(Remarks to the Author)
The authors have made substantial improvements to the manuscript. While they show that NCOR2 can regulate additional genes and pathways related to MHC class I, blocking the IFN pathway with an anti-IFN-g antibody fully reverses the effects of NCOR2 knockout on MHC class I expression and tumor metastasis (see Figure 6). This suggests that the immunological function of NCOR2 is primarily dependent on its regulation of the IFN pathway, which is the major finding from their previous publication.

Reviewer #4

(Remarks to the Author)
To this reviewer, the major flaw in this paper remains, in that there remains no causal relationship between NCOR2 recruitment and genomic binding, local epigenetic regulation and de novo transcriptional regulation of the MHC Class 1 locus, and certainly no evidence that any of the links suggested are genomically significant.
Briefly, NCOR2 will most likely bind to ~ 5000 high confidence sites across the murine or human genome, and although there is some evidence that the MYB/SANT motifs can allow NCOR2 to bind directly, most of its association with the genome is through recruitment by a transcription factor. If this event influences transcription, then this will be concomitant with altered histone modifications (e.g. loss of H3K27ac) possibly chromatin looping and then subsequent transcription initiation. As far as I can see none of these questions are in anyway addressed in the current study and therefore the link remains a correlation of unknown significance which may or may not be casual.
Compounding the correlative nature of the data is that of course, the breast cancer community will be very familiar with the idea that NCOR2 is recruited by nuclear receptors and other disease-relevant transcription factors in the context of cancer subtypes and disease progression. Therefore, the authors have not only to demonstrate this specific functional link between NCOR2 and MHC, but also demonstrate that for some unknown reason this incredibly potent link has remained undiscovered and that links between NCOR2 and say, nuclear receptors or NF-kB are not as significant as this one. That's a hard argument to make.
Finally, the bioinformatic approaches are poor.
1. It’s actually unclear if the NCOR2 cistrome studies were undertaken in triplicate and if the data are publically deposited
2. The enrichment analsyes is not described and therefore not reproducible
3. How many NCOR2 peaks were identified and how are they distributed across the genome, and how do they intersect with other known TNBC cistrome data?
4. Of the peaks identified (this reviewer is assuming 1000s of significant peaks were identified) how were they impacted by NCOR2 knockdown, in that were all peaks diminished or was there a skewing of events, which is probably most likely
5. Of the peaks identified where do the genes illustrate rank? More specifically, are the actual peaks illustrated the most

significant of all NCOR2 peaks detected (each gene may have more than one peak and picking the biggest one is not accurate)
6. Without these foundational data the presentation becomes dangerously close to data misrepresentation
7. Why use a scRNA-Seq data set and then use it like a bulk RNA-Seq data set? This is highly problematic given the sparseness of scRNA-Seq data. Of all the genes altered in the tumor subgroups where did NCOR2 and the other genes rank? To infer an inverse correlation requires statistical testing, and when done what genes are most negatively correlated and where does the relationship between NCOR2 and HLA-B, for example, rank?
8. Why not do these analyses in TCGA-BRCA or AURORA data sets where there are many more TNBC samples (with genomic ancestry data) to test these relationships? As an aside, across the whole TCGA-BRCA cohort the most significant inverse correlation of NCOR2 is with MRLP1 (spearman = -.408, qval = 8.5e-52), by contrast the most significant NCOR2 negative correlation with any of the indicated genes in the current study is with HLA-B (spearman = -.12, qval = 1.4e-4)

Version 1:

Reviewer comments:

Reviewer #4

(Remarks to the Author)
It is clear that the authors have made an earnest and serious effort to address the criticisms and as a result the manuscript is much improved. It is clear that NCOR2 exerts a potent biological effect in the cancer biology being studied, and also clearly some aspects that is centered on, for example, IFNg signaling in a NCOR2 dependent manner. This study then is an exciting addition to our understanding of where the epigenome and tumor biology interface in the context of the immune system.

Open Access This Peer Review File is licensed under a Creative Commons Attribution 4.0 International License, which permits use, sharing, adaptation, distribution and reproduction in any medium or format, as long as you give appropriate credit to the original author(s) and the source, provide a link to the Creative Commons license, and indicate if changes were made.
In cases where reviewers are anonymous, credit should be given to 'Anonymous Referee' and the source.
The images or other third party material in this Peer Review File are included in the article’s Creative Commons license, unless indicated otherwise in a credit line to the material. If material is not included in the article’s Creative Commons license and your intended use is not permitted by statutory regulation or exceeds the permitted use, you will need to obtain permission directly from the copyright holder.
To view a copy of this license, visit https://creativecommons.org/licenses/by/4.0/

REBUTTAL: We thank the reviewers for their constructive comments. We have conducted, as requested, additional studies to address all new concerns. We are confident that the reviewer(s) and the editor will now deem our revised manuscript suitable for publication in Nature Communication.

Reviewer #1 (Remarks to the Author):

The authors have addressed previous comments.

We are pleased that the reviewer is satisfied with our revised manuscript.

Reviewer #3 (Remarks to the Author):

The authors have made substantial improvements to the manuscript. While they show that NCOR2 can regulate additional genes and pathways related to MHC class I, blocking the IFN pathway with an anti-IFN-g antibody fully reverses the effects of NCOR2 knockout on MHC class I expression and tumor metastasis (see Figure 6). This suggests that the immunological function of NCOR2 is primarily dependent on its regulation of the IFN pathway, which is the major finding from their previous publication.

We are pleased that the reviewer is satisfied with our revised manuscript.

Reviewer #4 (Remarks to the Author):

To this reviewer, the major flaw in this paper remains, in that there remains no causal relationship between NCOR2 recruitment and genomic binding, local epigenetic regulation and de novo transcriptional regulation of the MHC Class 1 locus, and certainly no evidence that any of the links suggested are genomically significant.

Briefly, NCOR2 will most likely bind to ~ 5000 high confidence sites across the murine or human genome, and although there is some evidence that the MYB/SANT motifs can allow NCOR2 to bind directly, most of its association with the genome is through recruitment by a transcription factor. If this event influences transcription, then this will be concomitant with altered histone modifications (e.g. loss of H3K27ac) possibly chromatin looping and then subsequent transcription initiation. As far as I can see none of these questions are in anyway addressed in the current study and therefore the link remains a correlation of unknown significance which may or may not be casual.

We thank the reviewer for their astute critique. We agree that our initial data analysis and presentation in the last manuscript version was rudimentary. In this revised manuscript we have now addressed this issue. We enlisted Shivang Sharma who is a bioinformatics expert with experience analyzing Cut&Tag data set from the laboratory of Eugene Shenderov at Hopkins University. This included comprehensive normalization, statistical assessment, peak identification and validation. We concur that NCOR2 does indeed bind with high confidence to thousands of sites across the murine genome (which is what we assessed in this article). Please refer to our responses below.

Compounding the correlative nature of the data is that of course, the breast cancer community will be very familiar with the idea that NCOR2 is recruited by nuclear receptors and other disease-relevant transcription factors in the context of cancer subtypes and disease progression. Therefore, the authors have not only to demonstrate this specific functional link between NCOR2 and MHC, but also demonstrate that for some unknown reason this incredibly potent link has remained undiscovered and that links between NCOR2 and say, nuclear receptors or NF-kB are not as significant as this one. That's a hard argument to make.

We appreciate the reviewer's contention that the breast cancer community will be very familiar with the idea that NCOR2 is recruited by nuclear receptors and other disease-relevant transcription factors in the context of cancer subtypes and disease progression. We agree with the reviewer's comments. However, we respectfully point out that our experiments were not meant to be comprehensive with respect to NCOR2 gene targeting. Rather these studies serve to identify putative molecular mechanisms whereby NCOR2 regulates MHC class 1 molecule cell surface expression. We agree that many other pathways regulated by NCOR2 likely function in parallel. In our defense our cell biology studies do attest to the importance of NCOR2 regulation of the Tap genes. Nevertheless, we apologize for the superficial nature of our initial analysis and data presentation. We suggest that future experiments would need to be undertaken to comprehensively analyze our findings pertaining to the Tap genes through strategies including mutational manipulation and additional ChIP studies and HDAC modulation. We suggest that to comprehensively address some of this reviewer's comments go far beyond the scope of the current article and will be pursued in future work.

Thus, we contend that this new analysis confirms our initial observations that links do exist between NCOR2 and MHC regulation through the tap genes – as one putative mechanism. We agree that there are many other genes that NCOR2 likely regulates and have listed several in the supplemental data section for examination. We respectfully point out that this does not detract from our observation and conclusion that NCOR2 does appear to regulate cell surface levels of MHC class 1 molecules in an INF gamma dependent manner. An important issue that may differentiate our current studies from work conducted by other laboratories studying hormone receptor interactions is that the breast tumor lines that were used to conduct the studies reported in this article do not express ER or PR receptors. Perhaps this is one reason why this specific NCOR2 relationship has yet to be identified. Clearly, once the work has been published the validity of our experimental results will be examined by other researchers who can experimentally examine the relationship between NCOR2 and MHC class 1 cell surface presentation, and either agree or disagree with the findings presented in this manuscript. Below we outline our responses to the critiques raised by this reviewer.

Finally, the bioinformatic approaches are poor.

1. It's actually unclear if the NCOR2 cistrome studies were undertaken in triplicate and if the data are publically deposited

We apologize to the reviewer for the rudimentary nature of the data presented in our earlier revised manuscript. We have recruited a bioinformatics expert familiar with Cut&Tag analysis who conducted a statistically rigorous analysis of our findings. NOTE: As required the data will be made available at Gene Expression Omnibus (GEO) and the accession number will be open for public downloads upon publication.

Below we show the sample size for each category.

Treatment	Knockdown	N
Untreated	NCOR2	4
Untreated	EGFP	2
Untreated	Ctrl (IgG Control)	1
Treated	NCOR2	4
Treated	EGFP	4
Treated	Ctrl (IgG Control)	4

2. The enrichment analsyes is not described and therefore not reproducible

Regarding enrichment analysis: **MACS3 is used with filtered high-quality reads for peak enrichment. Briefly, MACS3 builds pileups using sample bam and IgG control. MACS3 assumes that IgG controls comprised predominantly of background that follows normal distribution. A peak is enriched in the sample if the pileup at a genomic location is significantly higher than the control pileup. A False Positive Detection Rate (FDR) of 0.01 is used for peak enrichment analysis. A more detailed explanation of enrichment strategy is provided in the Methods section.**

Regarding Quality control analysis: **Extensive read quality assessment and statistics have been summarized and reported in the multiqc file provided as supplementary data.**

3. How many NCOR2 peaks were identified and how are they distributed across the genome, and how do they intersect with other known TNBC cistrome data?

Genomic distributions of peaks for every sample including controls are available in files with the naming convention <Sample_Name>.markdup.q30_peaks.xls

Approximately 51,000 significant peaks were identified across all samples. We did not restrict ourselves to transcription start sites at the beginning of a gene but rather performed a completely unbiased peak enrichment analysis. Hence, many peaks were identified at the intronic regions in the middle of genes. Also, several visually appearing to be single peak were subdivided for independent analysis of the subpeaks.

The peaks were distributed across all the chromosomes of mouse genome with marginal distribution on the X chromosome and negligible distribution on the y chromosome. There was also a very sparse distribution of peaks on the mitochondrial chromosome

The raw counts data for peaks in each sample have now been provided as a supplementary file and included in the revised manuscript.

4. Of the peaks identified (this reviewer is assuming 1000s of significant peaks were identified) how were they impacted by NCOR2 knockdown, in that were all peaks diminished or was there a skewing of events, which is probably most likely

The peak enrichment strategy set a false discovery rate at 0.01. Out of the ~51000 peaks that were identified, 15,786 peaks showed a significant dysregulation upon knockdown. Out of these only 27 of them were marginally upregulated in NCOR2 knockdown while the remaining were downregulated.

An integrated raw counts assessment for each peak in every sample has now been provided in the supplementary data section in the revised manuscript.

5. Of the peaks identified where do the genes illustrate rank? More specifically, are the actual peaks illustrated the most significant of all NCOR2 peaks detected (each gene may have more than one peak and picking the biggest one is not accurate)

The Tap genes illustrated in the main figures illustrate a strong statistical significance Tap1 (log2FC = 1.2, p-value < 0.05, Rank = 15527), Tap2 (log2FC = 1.46, p-value < 0.01, Rank = 8608; log2FC = 1.5, p-value < 0.0001, Rank = 801) and Tapbp (log2FC = 0.8, p-value < 0.01, Rank = 8011) where the fold change values represent overexpression in EGFP control vs NCOR2 knockdown. However, these genes did not top the list of significant genes that were dysregulated due to NCOR2 knockdown. The top genes comprised genes like Sos2, Adamts7, Vwa3b, Ccdc126, etc. We did not filter out to the biggest peak within a gene and all the peaks were reported irrespective of their location relative to known genes in the mouse genome.

6. Without these foundational data the presentation becomes dangerously close to data misrepresentation

Once again, we apologize to this reviewer regarding the superficial data analysis we presented regarding the Cut&Tag studies in our earlier version of this manuscript. Our collaborators conducted a statistical rigorous analysis of the Cut&Tag data sets and outlined the strategies they used for their work flow.

7. Why use a scRNA-Seq data set and then use it like a bulk RNA-Seq data set? This is highly problematic given the sparseness of scRNA-Seq data. Of all the genes altered in the tumor subgroups where did NCOR2 and the other genes rank? To infer an inverse correlation requires statistical testing, and when done what genes are most negatively correlated and where does the relationship between NCOR2 and HLA-B, for example, rank?

Pseudo bulk analysis of single-cell RNA sequencing data is a common approach to identify biomarkers, therapeutic targets and genomic stratification, and in some instances they even outperform single-cell based differential expression methods [1]. Several methodologies have been developed to streamline the pseudo bulk analysis of single cell data [2][3]. It is appropriate and possible to perform pseudo bulk analysis when the cell counts are sufficiently large to overcome the problems associated with sparsity of single-cell data and the analysis wants to obtain a pure subpopulation of a cell type to inform the analysis. Conversion of single-cell RNA sequencing samples into pseudo bulk eliminates the issues associated with sparsity including but not limited to noisy expression patterns and lower interpretability.

[1]: https://www.nature.com/articles/s41467-021-25960-2
[2]: https://www.nature.com/articles/s41467-022-35519-4
[3]: https://academic.oup.com/bioinformatics/article/40/8/btae498/7730101

We respectfully point out that the aim of scRNA-seq data analysis was to explore the correlation between NCOR2 and MHC associated genes in TNBC samples. Although differential expression of genes in TNBC vs other groups can provide useful insights, we maintain that this additional analysis is unnecessary and is beyond the scope of this work. Similarly, we believe that a genome wide correlation rank of genes with NCOR2 fails to answer the questions being investigated in this work and hence does not fall within the scope of this analysis.

Below are the heatmaps for (i) spearman correlation values between NCOR2, HLA-A, HLA-B and B2M, and (ii) p-values for spearman correlations between each pair of genes.

(i) Spearman correlation heatmap

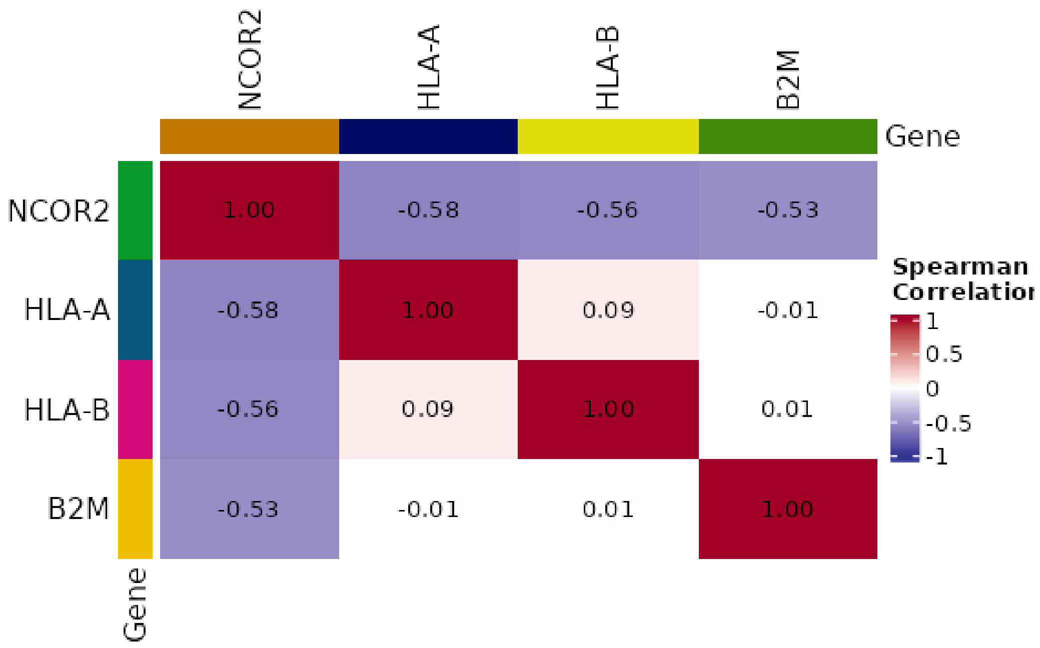

(ii) p-value heatmap

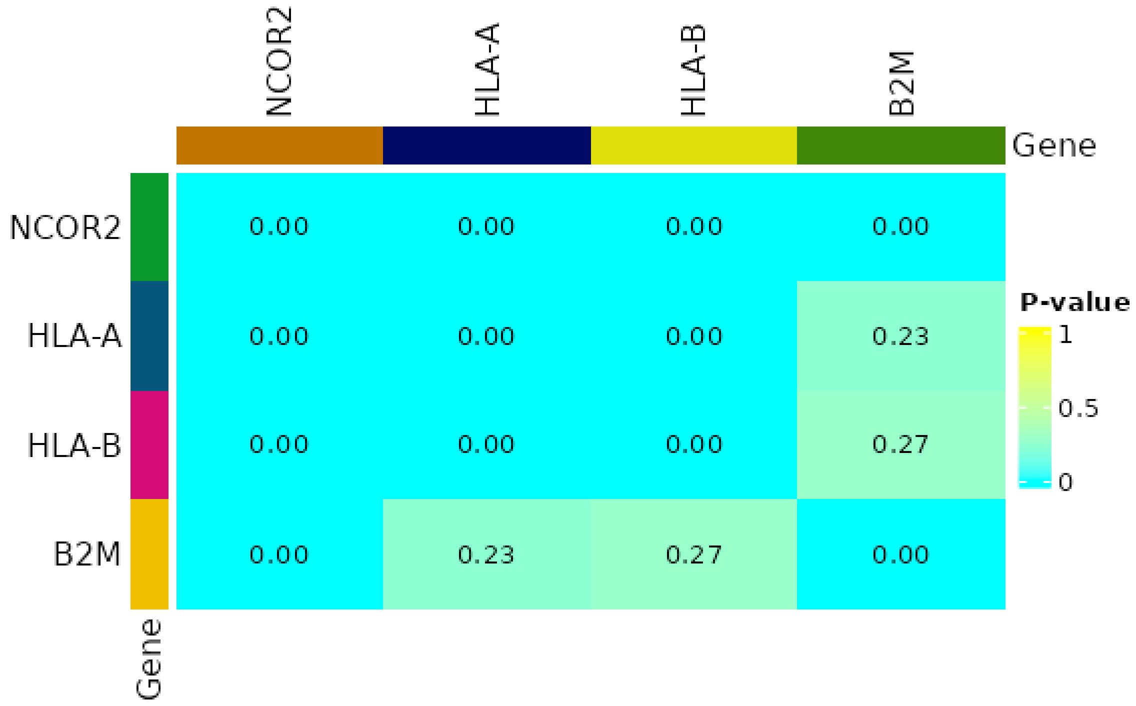

8. Why not do these analyses in TCGA-BRCA or AURORA data sets where there are many more TNBC samples (with genomic ancestry data) to test these relationships? As an aside, across the whole TCGA-BRCA cohort the most significant inverse correlation of NCOR2 is with MRLP1 (spearman = -.408, qval = 8.5e-52), by contrast the most significant NCOR2 negative correlation with any of the indicated genes in the current study is with HLA-B (spearman = -.12, qval = 1.4e-4)

We agree with the reviewer that the datasets referred to above would certainly provide the advantage of a higher sample size. However, TCGA is comprised predominantly of primary samples with very few metastatic and even fewer TNBC samples. Moreover, the MET500 dataset comprises 159 metastatic breast cancer datasets. Hence, we believe the sample size in MET500 is statistically sufficient to test our hypotheses.

Transcriptomics datasets are highly susceptible to batch effects induced at library preparation and sequencing. Hence, a parallel comparison of differential regulation of genes without an exhaustive batch correction should be avoided. Moreover, the TCGA-BRCA is comprised primarily of primary tumors as compared with the MET500 cohort which by contrast is comprised of only metastatic tumors. This is yet an additional reason why we contend that the comparison between the two datasets as suggested by this reviewer should be avoided.

Reviewer #4 (Remarks to the Author):

It is clear that the authors have made an earnest and serious effort to address the criticisms and as a result the manuscript is much improved. It is clear that NCOR2 exerts a potent biological effect in the cancer biology being studied, and also clearly some aspects that is centered on, for example, IFNg signaling in a NCOR2 dependent manner. This study then is an exciting addition to our understanding of where the epigenome and tumor biology interface in the context of the immune system.

We thank the reviewer for their comments on our efforts and the significance of our work. We are pleased that the reviewer is satisfied with our attempts to address criticisms.
